# Exploring DNA variant segregation types in pooled genome sequencing enables effective mapping of weeping trait in *Malus*

**DOI:** 10.1093/jxb/erx490

**Published:** 2018-01-29

**Authors:** Laura Dougherty, Raksha Singh, Susan Brown, Chris Dardick, Kenong Xu

**Affiliations:** 1 Horticulture Section, School of Integrative Plant Science, Cornell University, USA; 2 USDA-ARS Appalachian Fruit Research Station, USA

**Keywords:** DNA variants, *Malus*, pooled genome sequencing, RNA-seq, segregation types, weeping

## Abstract

To unlock the power of next generation sequencing-based bulked segregant analysis in allele discovery in out-crossing woody species, and to understand the genetic control of the weeping trait, an F_1_ population from the cross ‘Cheal’s Weeping’ × ‘Evereste’ was used to create two genomic DNA pools ‘weeping’ (17 progeny) and ‘standard’ (16 progeny). Illumina pair-end (2 × 151 bp) sequencing of the pools to a 27.1× (weeping) and a 30.4× (standard) genome (742.3 Mb) coverage allowed detection of 84562 DNA variants specific to ‘weeping’, 92148 specific to ‘standard’, and 173169 common to both pools. A detailed analysis of the DNA variant genotypes in the pools predicted three informative segregation types of variants: <lm×mm> (type I) in weeping pool-specific variants, and <lm×ll> (type II) and <**h**k×**h**k> (type III) in variants common to both pools, where the first allele is assumed to be weeping linked and the allele shown in bold is a variant in relation to the reference genome. Conducting variant allele frequency and density-based mappings revealed four genomic regions with a significant association with weeping: a major locus, *Weeping* (*W*), on chromosome 13 and others on chromosomes 10 (*W2*), 16 (*W3*), and 5 (*W4*). The results from type I variants were noisier and less certain than those from type II and type III variants, demonstrating that although type I variants are often the first choice, type II and type III variants represent an important source of DNA variants that can be exploited for genetic mapping in out-crossing woody species. Confirmation of the mapping of *W* and *W2*, investigation into their genetic interactions, and identification of expressed genes in the *W* and *W2* regions provided insight into the genetic control of weeping and its expressivity in *Malus*.

## Teaser

Identifying and exploiting three informative DNA variant segregation types in pooled genome sequencing analysis of the weeping phenotype in *Malus* empowers an effective genetic mapping strategy in out-crossing woody species.

## Introduction

The development of next generation sequencing (NGS) technologies has revolutionized approaches in genetics and genomics (Mardis, 2008; Schuster, 2008; Quail *et al.*, 2012). Implementing NGS technology has enabled whole genome sequencing in bulked segregant analysis (BSA), a methodology (Giovannoni *et al.*, 1991; Michelmore *et al.*, 1991) widely used in genetic mapping by analysing two pools of genomes of contrasting phenotypes (Lister *et al.*, 2009; Schneeberger *et al.*, 2009). A major advantage of the approach is that it can simultaneously allow gene/quantitative trait locus (QTL) mapping, fine mapping, and causal mutation identification. After the first successful demonstrations in Arabidopsis (Lister *et al.*, 2009; Schneeberger *et al.*, 2009), the approach has been adapted in many other species, such as legumes (Sandal *et al.*, 2012), rice (Abe *et al.*, 2012), wheat (Trick *et al.*, 2012), arthropods (Van Leeuwen *et al.*, 2012), zebrafish (Obholzer *et al.*, 2012), and peach (Dardick *et al.*, 2013). Similar analyses using RNA-seq data were reported in maize and zebrafish (Liu *et al.*, 2012; Hill *et al.*, 2013; Miller *et al.*, 2013). A few of the latest examples of using pooled genome sequencing analyses to identify important genes in plants include the lettuce thermotolerant seed germination gene *ABA1/ZEP* (Huo *et al.*, 2016), the glycerol-3-phosphate acyltransferase gene *GPAT6* crucial in tomato fruit cutin biosynthesis (Petit *et al.*, 2016), and the gibberellic acid receptor *PpeGID1c* gene for brachytic dwarfism in peach (Hollender *et al.*, 2016). In addition, the approach has been extended to mapping genetic variants associated with DNA methylation (Kaplow *et al.*, 2015) and genome-wide association studies (Yang *et al.*, 2015).

Various terms have been used to describe the application of NGS-enabled whole genome sequencing in BSA, such as mapping-by-sequencing (Hartwig *et al.*, 2012), whole genome sequencing (Sarin *et al.*, 2008; Leshchiner *et al.*, 2012; Schneeberger, 2014), pool-seq (Kofler *et al.*, 2011), MutMap (Abe *et al.*, 2012), QTL-seq (Takagi *et al.*, 2013), pnome (Dardick *et al.*, 2013), and others. Regardless of terminology, the basic ideas and principles behind the pooled genome sequencing approach are similar, i.e. the genome pool from individuals with a trait of interest would have more abundant DNA molecules carrying the causal variants than the genome pool from those without the trait. As a result, the frequency of the causal variant or the linked variants is expected to be different from that in unlinked regions. In the case of a dominant trait in the BC_1_ population, the causal variant frequency is expected to be *ca* 50% in the pool with the trait, whereas the frequency in the pool without the trait will be *ca* zero. The frequency of DNA variants towards both directions from the causal variant will progressively become lower than 50%, i.e. a causal mutation is most likely under the peak of DNA variant frequency in the pool carrying the trait of interest in this example.

To facilitate data analysis, several analytical software packages have been developed, such as SHOREmap (Schneeberger *et al.*, 2009; Sun and Schneeberger, 2015), CloudMap (Minevich *et al.*, 2012), SNPtrack (Leshchiner *et al.*, 2012), MegaMapper (Obholzer *et al.*, 2012), MMAPPR (Hill *et al.*, 2013), EXPLoRA and EXPLoRA-Web (Duitama *et al.*, 2014; Pulido-Tamayo *et al.*, 2016), and GIPS (Hu *et al.*, 2016). These packages are helpful tools for pooled genome sequencing data analysis for many model species from which they were developed. However, efforts are needed to make them more user-friendly and/or to broaden their application range to cover non-model species or species without a high-quality reference genome. In addition, accurate calling of variants remains challenging as a considerable fraction of variants that are false appear to be inherent to commonly used variant callers (Huang *et al.*, 2015; Ribeiro *et al.*, 2015).

A number of mapping strategies for positioning causal variants using pooled genome sequencing data have been proposed and demonstrated with successful applications, such as variant scarcity or density mappings (Schneeberger *et al.*, 2009; Zuryn *et al.*, 2010), variant discovery mapping (Minevich *et al.*, 2012), SNP index (Abe *et al.*, 2012), bulk segregant linkage mapping (Obholzer *et al.*, 2012), delta SNP index mapping (Fekih *et al.*, 2013; Takagi *et al.*, 2013), SNP ratio mapping (SRM) (Lindner *et al.*, 2012), mutant allele frequency (MAF), allelic distance (AD), and homozygosity mapping (Schneeberger, 2014). These mapping strategies largely can be attributed to the use of three major parameters, namely variant allele frequency, variant density, and variant distance (allelic distance). It should be possible in principle to conduct pooled genome sequencing-based genetic mapping studies in *Malus* species although the DNA variants are of complex segregation patterns and the phase is often unknown due to their heterogeneously heterozygous genome.

The weeping growth habit in woody species represents a unique form of tree architecture and has been an essential element in landscape aesthetics. Compared with standard trees with branches that grow mostly upward with certain angles, weeping tree branches grow downward. In *Malus*, the weeping (pendulous) phenotype is found in *M. domestica*, such as cv ‘Elisa Ratkee’, but it is more frequently seen in crabapples for ornamental purpose, such as ‘Exzellenz Thiel’, ‘Red Jade’, *M. baccata* ‘Gracilis’, ‘Cheal’s Weeping’, and ‘Louisa’. ‘Red Jade’ is believed to be derived from an open pollinated seedlings of ‘Exzellenz Thiel’, which was selected from the cross *M. prunifolia* ‘Pendula’ × *M. floribunda* (Brown *et al.*, 2004). The weeping phenotype in *M. baccata* ‘Gracilis’ is controlled by a single dominant allele, called *Weeping* (*W*), based on an inheritance study conducted in two small populations of 28 seedlings derived from *M. baccata* ‘Gracilis’ (Sampson and Cambron, 1965; Brown, 1992; Alston *et al.*, 2000). In a population of 98 seedlings from the cross ‘Wijcik McIntosh’ (columnar) × ‘Red Jade’ (weeping), the weeping and columnar phenotypes segregated independently despite intermediates expressing both phenotypes, i.e. columnar at the top and weeping at the bottom (Just, 2001). Two DNA markers (GD147 and CS44_1150_) linked to the weeping phenotype were also identified although the trait was not mapped (Just, 2001). The allelic relationships between ‘Red Jade’ and other weeping crabapples including *M. baccata* ‘Gracilis’, ‘Cheal’s Weeping’, and ‘Louisa’ are unknown.

Cultivated apple tree architecture has been categorized into four types based on the overall growth habit: columnar (e.g. ‘Wijcik McIntosh’), spur (‘Starkrimson’), standard (‘Golden Delicious’), and weeping (‘Granny Smith’) (Lespinasse and Delort, 1986; Lespinasse, 1992; Costes *et al.*, 2006; Pereira-Lorenzo *et al.*, 2009; Höfer *et al.*, 2013). However, ‘Granny Smith’ trees grow branches similar to a standard tree. Their weeping-like trait is due to the bending of branches that bear fruit at the tips. Such tip-bearing-caused bending of branches is different from weeping caused by the downward growth of branches, and is distinct from the trait mapped in this report. A better understanding of the genetic architecture responsible for the weeping trait in *Malus* would provide important insight into directional growth of shoot meristems in woody species.

In this study, based on a detailed analysis of DNA variant genotypes in the weeping and standard pools and their possible segregation types, an effective strategy was devised to target three informative segregation types of variants: <**l**m×mm> (type I) in weeping pool-specific variants, and <**l**m×**ll**> (type II) and <**h**k×**h**k> (type III) in variants common to both pools. Note that the first allele is designated weeping linked from ‘Cheal’s Weeping’ and the alleles in bold represent a DNA variant in relation to the apple reference genome. Although type I variants are the most straightforward for mapping, types II and III variants performed better in mapping the weeping trait, highlighting their utility in pooled genome sequencing-based genetic mapping. To the best of our knowledge, this is the first report on identifying and exploiting three informative segregation types of DNA variants in pooled genome sequencing analysis for genetic mapping in an out-crossing woody species of highly heterozygous genome.

## Materials and methods

### Plant material and growth habit evaluation

**Fig. 1.**
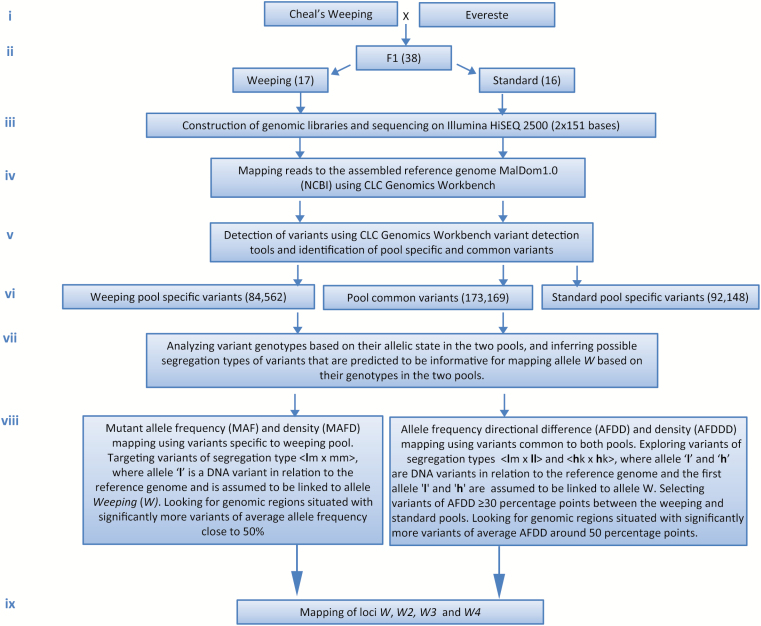
A flowchart illustrating the major steps in MAFD and AFDDD mappings of the weeping phenotype. (This figure is available in color at *JXB* online.)

Three F_1_ populations segregating for weeping growth habit were used for genetic mapping of the trait. The first comprised 38 seedling trees (8 years old) from the cross ‘Cheal’s Weeping’ × ‘Evereste’ (Fig. 1i, ii); the second was developed from NY-051 × ‘Louisa’ of 140 progeny; and the third was derived from NY-011 × NY-100 consisting of 39 individuals. The progeny in the second and third populations were 2 years old. ‘Cheal’s Weeping’ and ‘Louisa’ are weeping crabapple cultivars. NY-100 is a weeping selection from the progeny of ‘Red Jade’, another weeping crabapple cultivar. The relatedness of ‘Cheal’s Weeping’, ‘Louisa’, and ‘Red Jade’ is unknown. ‘Everest’, NY-051 and NY-011 are crabapples of standard growth habit. The populations were planted in a research orchard of Cornell University in Geneva, NY, USA. Evaluation of growth habits was conducted by visual observation and seedling trees were categorized into weeping, weeping-like, standard, standard-like and intermediate (Supplementary Table 1Supplementary Fig. S1 at *JXB* online).

### Construction and sequencing of genomic DNA pools

Genomic DNA samples were prepared from young leaf tissues as previously described (Wang *et al.*, 2012) and were quantified using Qubit dsDNA BR Assay Kit on a Qubit 3.0 fluorometer (Invitrogen, Carlsbad, CA, USA). An equal amount of DNA (300 ng) from each of the 17 weeping (-like) and 16 standard progeny in population ‘Cheal’s Weeping’ × ‘Everest’ was combined into a weeping pool and a standard pool, respectively (Fig. 1ii). Genomic DNA libraries of target insert size of 500 bp were constructed from each of the two genomic DNA pools using an Illumina (San Diego, CA, USA) TruSeq DNA PCR-Free Library Preparation Kit, and then paired-end (2 × 151 bp) sequenced on an Illumina HiSEQ 2500 platform (Fig. 1iii) at the Genomics Facility of Cornell University (Ithaca, NY, USA).

### Mapping of reads to the apple reference genome

The assembled apple ‘Golden Delicious’ genome MalDom1.0 (NCBI accession GCA_000148765.1, annotation release 100, June 2014) (Velasco *et al.*, 2010), which comprises 17 chromosomes with a total size of 526 197 889 bp, was used as reference. Mapping of the Illumina sequencing reads onto the reference genome was conducted in the weeping and standard pools using the software CLC Genomics Workbench (v7.5, CLCBio, Cambridge, MA, USA). The mapping parameters and settings were similar to previously described (Bai *et al.*, 2014), i.e. the minimum length fraction was 0.8 and the minimum similarity was 0.98 (Fig. 1iv; Supplementary Table 1Supplementary Table S1).

### Detection and analysis of DNA variants

In the weeping or standard pool, detection of DNA variants was conducted using the fixed ploidy (2×) variant detection tool embedded in CLC Genomics Workbench (Fig. 1v). Variant frequency was calculated automatically based on the total number of reads aligned at the region. To capture as many variants as possible initially, the minimum coverage was ten and the minimum count of variant reads was two. The variants were filtered through a series of filters to remove variants that are reference alleles, hyperallelic, homopolymers, and/or called when reference is an ambiguous base, such as M, R, W, S, Y, and K (Supplementary Table 1Supplementary Table S2). Variants specific to either pool and variants common to both pools were identified by direct comparison between the set of variants identified in the weeping pool and those in the standard pool using CLC Genomic Workbench (Fig. 1vi). To minimize false positive variants prior to mapping the trait, these pool-specific and common variants were filtered again by another set of filters: read coverage ≥20, forward/reverse reads balance 0.25–0.5, number of reads with unique start positions ≥5 (Supplementary Table 1Supplementary Table S2).

DNA variants specific to the weeping pool and those common to both pools were considered genetically informative for mapping allele *weeping* (*W*), whereas variants specific to the standard pool were used as control (Fig. 1vi). Variants of allele frequency ranged from 15% to 80% were classed heterozygous while those >80% were classed as homozygous (Fig. 1vii).

### Mutant allele frequency and density mapping

Mutant allele frequency and density (MAFD) mapping is an adaptation of mutant allele frequency (MAF) mapping described previously (Schneeberger, 2014) by integration of a second parameter, variant density. It employs the weeping pool-specific variants of segregation type <**l**m×mm> (type I), where allele ‘**l**’ is a variant in relation to the reference genome and is assumed to be linked to allele *W*. In practice, the focus is on examining which genomic regions that might be situated with variants of average allele frequency close to 50% and how the variants were distributed along the genome (Fig. 1viii).

### Allele frequency directional difference and density mapping

Allele frequency directional difference and density (AFDDD) mapping explores two groups of variants common to both pools. The first group is of segregation type <**l**m×**ll**> (type II), where the first allele ‘**l**’ is also a variant and assumed to be linked to weeping. The expected average variant allele frequency is 100% in the weeping pool and 50% in the standard pool in the *W* region. The second group of variants is of segregation type <**h**k×**h**k> (type III), where ‘**h**’ is a variant and the first allele is linked to allele *W*. Under this scenario, the expected average allele frequency at the *W* locus is 75% in the weeping pool and 25% in the standard pool. The allele frequency directional difference (AFDD) threshold in AFDDD mapping is AFDD≥30 percentage points. In this case, the goal is to look for the genomic regions that would situate with significantly more variants of average AFDD close to 50 percentage points (Fig. 1viii).

### Standard score (*z*) test

Genome-wide distribution of DNA variants of the three segregation types <**l**m×mm>, <**l**m×**ll**>, and <**h**k×**h**k> is assumed to be about even. In both MAFD and AFDDD mappings, if a genomic region is observed with a significant increase from the mean in variant density, the region is thought to be associated with the weeping phenotype. The significance test was conducted by standard score (*z*), which is calculated by the formula *z*=(*X−*μ)/σ, where *X* is observed variant density (variants per Mb); μ is mean variant density in the data set; and σ is standard deviation of the mean (μ). The cut off is *z*=2.6, *P*=0.01 (two-tailed confidence level).

### Marker development

SSR markers were identified and developed from the apple reference genome sequence in the *W* and *W2* regions as previously described (Xu *et al.*, 2012). The primer sequence information and their approximate physical location in the genome are listed (Supplementary Table 1Supplementary Table S3). Polyacrylamide gel electrophoresis of SSR markers were conducted as detailed previously (Wang *et al.*, 2012).

### Sanger DNA sequencing

For confirmation of the variants of segregation types<**h**k×**h**k> and <**l**m×**ll**>, four genomic segments in the *W* region were PCR amplified with specifically designed PCR primers (Supplementary Table 1Supplementary Table S3) and the PCR products were sequenced directly by an ABI 3730XL DNA sequencer at the Cornell Genomics Facility Center.

### RNA-seq and qRT-PCR analyses

Total RNA samples were isolated from actively growing shoot tip tissues of four weeping and four standard progeny individually from the population ‘Cheal’s Weeping’ × ‘Evereste’ using Qiagen Plant RNA Isolation Kit (Germantown, MD, USA). The Isolated RNA samples were pooled by phenotypes, forming a weeping and a standard RNA pool, respectively. Construction of RNA-seq libraries for the weeping pool and the standard pool were conducted similarly to that described earlier (Bai *et al.*, 2014). Single-end sequencing of read length 76 bp was performed on an Illumina NextSEQ 500 platform. RNA-seq reads were mapped to the improved or the latest version of the apple reference transcriptomes (Bai *et al.*, 2014; Daccord *et al.*, 2017) using CLC Genomics Workbench. Validation of RNA-seq analysis was performed by qRT-PCR assays on ten selected genes in the four weeping and four standard progeny. The qRT-PCR procedures were similar to what was described previously (El-Sharkawy *et al.*, 2015), and the primers, including those for the reference gene *MdActin*, are listed (Supplementary Table 1Supplementary Table S4).

### BLAST-based dot matrix analysis

BLAST-based dot matrix analysis was performed using the BLAST tool for aligning two sequences, which is available at the NCBI website (https://www.ncbi.nlm.nih.gov/). The input of query sequences was limited to the regions of *W*, *W2*, *W3*, and *W4* in accessions CM001038.1 (Chr13), CM001035.1 (Chr10), CM001041.1 (Chr16), and CM001030.1 (Chr5) from the first version of the apple reference genome (Velasco *et al.*, 2010), respectively. The input of subject sequences was correspondingly the entire sequences of chromosomes 13 (CM007879.1), 10 (CM007876.1), 16 (CM007882.1), and 5 (CM007871.1) from the new apple reference genome (Daccord *et al.*, 2017). The regions of similarity were visualized by the dot matrix tool available at the NCBI website as well.

## Results

### Segregation of weeping growth habit

**Fig. 2.**
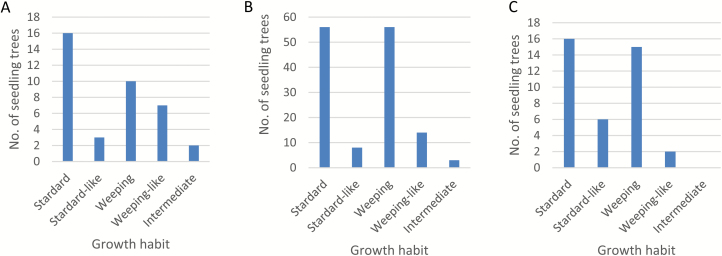
Phenotypic evaluation of growth habit in populations ‘Cheal’s Weeping’ × ‘Evereste’ (A), NY-051 × ‘Louisa’ (B), and NY-011 × NY-100 (C) segregating for weeping phenotype. Chi-square tests (excluding the intermediates) showed that the segregation of weeping (-like) and standard (-like) growth habits fit the 1:1 ratio in all the three populations: ‘Cheal’s Weeping’ × ‘Evereste’, χ^2^=0.1111, *P*=0.74; NY-051 × ‘Louisa’, χ^2^=0.2687, *P*=0.60; and NY-011 × NY-100, χ^2^=0.4100, *P*=0.52. (This figure is available in color at *JXB* online.)

In population ‘Cheal’s Weeping’ × ‘Evereste’, consisting of 38 individuals, 19 were scored standard (16) or standard-like (3) in growth habit, 17 were weeping (10) and weeping-like (7), and two were intermediate (Fig. 2A). In population NY-051 × ‘Louisa’, comprising 140 seedling trees, 64 were scored standard (56) or standard-like (8) in growth habit, 70 were weeping (56) and weeping-like (14), and three were intermediate (Fig. 2B). The remaining three were dead or too weak for evaluation. In the NY-011 × NY-100 population, 22 individuals were noted as standard (16) and standard-like (6), whereas 17 were observed as weeping (15) and weeping-like (2) (Fig. 2C). Chi-square tests (excluding the intermediates) showed that the segregation of weeping (-like) and standard (-like) growth habits fit the 1:1 ratio in all three populations (*P*=0.52–0.74), suggesting that the weeping phenotype is largely a dominant trait controlled by a major locus, presumably *W*. Thus parents ‘Cheal’s Weeping’, ‘Louisa’, and NY-100 are of genotype *Ww* at the *W* locus, and ‘Evereste’, NY-051, and NY-011 are of genotype *ww*. The presence of individuals of less typical weeping and standard phenotype and intermediates in these populations suggests other modifying factors may exist.

### Pooled genome sequencing analysis and identification of DNA variants

Illumina sequencing generated 140742316 and 157357078 paired-end raw reads (2 × 151 bp) for the weeping and standard genome pools, respectively (NCBI accession SRP094968). After removing 7425504 (5.0%) low quality reads in the weeping pool and 7917860 (5.3%) in the standard pool, the cleaned 133316812 (27.1× the reference genome of 742.3 Mb in weeping pool) and 149439218 (30.4× standard) reads (Supplementary Table 1Supplementary Table S1) were used for alignment against the reference genome. The mapped reads were 57639266 for weeping and 66798158 for standard, accounting for 43.2% and 44.7%, and covering 16.5× and 19.2× the assembled reference genome (526.2 Mb), respectively (Supplementary Table 1Supplementary Table S1).

Using the variant detection tool of CLC Genomics Workbench, a total of 2700059 variants in weeping and 2946289 in standard pools were detected. The number of variants of non-reference allele was 1306887 (single nucleotide variant (SNV): 88.5%) and 1380503 (SNV: 87.8%) in weeping and standard pools, respectively (Supplementary Table 1Supplementary Table S2). Comparing the non-reference variants between the two pools identified 498386 unique to the weeping pool, 573589 unique to the standard pool, and 799089 in common. To use more reliable variants, another set of filters was applied (Supplementary Table 1Supplementary Table S2), leading to 84562 variants specific to the weeping pool, 92148 specific to the standard pool, and 173169 common to both pools, which constitute the primary datasets of variants for mapping the weeping trait (Supplementary Table 1Supplementary Table S2; Fig. 1vi). For an overview of these variants, the distributions according to their genotypes, allele frequencies and home chromosomes are shown in Supplementary Table 1Supplementary Figs S2 and Supplementary Table 1S3.

### Inferring segregation types of variants

‘Cheal’s Weeping’ × ‘Evereste’ is a cross between two heterogeneously heterozygous diploid parents. In such crosses, at least six segregation types are possible for a given DNA variant if phase is not considered, including <ab×cd>, <ef×eg>, <hk×hk>, <lm×ll>, <nn×np>, and <qq×qq>, where each letter stands for one of the four DNA bases (A, C, G, and T) in SNVs, or an allele of other DNA variant types. To be informative for mapping allele *W* in pooled genome sequencing analysis, variants must have: (i) a heterozygous genotype in ‘Cheal’s Weeping’; (ii) a dense coverage throughout the genome; and (iii) a segregation type segregating for a unique allele frequency in the weeping pool or a large difference in allele frequency between the weeping and standard pools so that discrimination is possible. It is expected that segregation types <nn×np> and <qq×qq> will not be informative based on criterion (i). Since the most abundant variants are SNVs involving two alleles, variants of segregation types <ab×cd> and <ef×eg>, which segregate for four and three alleles, respectively, likely would be much less frequent. Therefore, the remaining two segregation types, <hk×hk> and <lm×ll>, are predicted to be informative.

**Fig. 3.**
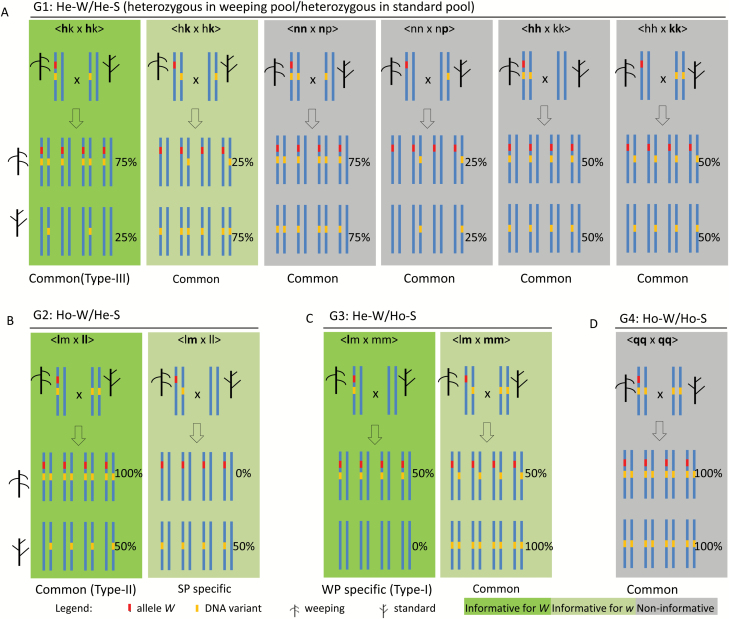
Schematic representations of the segregation of DNA variants linked to allele *W* in either phase under varying segregation types inferred for four of the five variant genotype groups: G1 (A), G2 (B), G3 (C), and G4 (D). Each segregation type is illustrated in a colored rectangle that includes the two parents at the top, four representative weeping progeny in the middle, and four standard progeny at the bottom. The long vertical lines in blue represent the chromosomal segment harboring *W*. The red and orange short vertical lines represent allele *W* and DNA variants in relation to the reference genome, respectively. The tree-like drawings with upward and downward ‘branches’ indicate standard and weeping tree phenotypes, respectively. The expected allele frequency (%) of DNA variants in the weeping and standard pools is given accordingly. In each segregation type denotation, the allele at the first position is designated as being linked to weeping phenotype in the seed parent ‘Cheal’s Weeping’ (e.g. letter ‘l’ in <**l**m×mm>), and those in bold are DNA variants in relation to the apple reference genome (e.g. letters ‘l’ in <**l**m×**ll**>). Segregation types informative for mapping allele *W* are shown in green rectangles (See Supplementary Table S5 for more details). ‘Common’, variants common to both pools; SP, standard pool; WP, weeping pool.

To develop an effective approach for genetic mapping of the weeping phenotype, the genotypes of the 173169 variants common to both pools were compared based on their allelic state observed in the weeping and standard pools (Fig. 1vii), leading to five genotype groups: G1: heterozygous in weeping (He-W)/heterozygous in standard (He-S); G2: homozygous in weeping (Ho-W)/He-S; G3: He-W/homozygous in standard (Ho-S); G4: Ho-W/Ho-S; and G5: ‘Complex’ for those of complex genotypes involving four or three different DNA bases (i.e. three or two DNA variants in relation to the reference), presumably caused by segregation type <ab×cd> or <ef×eg> (Supplementary Table 1Supplementary Table S5; Fig. 3; Supplementary Table 1Supplementary Fig. S4). G1 is the largest group, of 144558 (83.5%) variants, whereas the G2 and G3 groups are of 5353 (3.1%) and 2104 (1.2%) variants, respectively. The G4 and G5 groups had 16963 (9.8%) and 4191 (2.4%) variants, respectively (Supplementary Table 1Supplementary Table S5). The variants specific to the standard pool fall into group G2 and those specific to the weeping pool into G3.

Inferring segregation types conceivably responsible for the observed G1–G5 identified at least 12 possible segregation types when the phase of variants was considered (Supplementary Table 1Supplementary Table S5; Fig. 3; Supplementary Table 1Supplementary Fig. S4). Further analysis concluded that only segregation types <**l**m×mm> (type I) for G3, <**l**m×**ll**> (type II) for G2, and <**h**k×**h**k> (type III) for G1 are informative for mapping of *W*, where the alleles at the first position are designated as being linked to the weeping phenotype in the seed parent ‘Cheal’s Weeping’ and those in bold are polymorphic variants in relation to the apple reference genome (Supplementary Table 1Supplementary Table S5; Fig. 3; Supplementary Table 1Supplementary Fig. S4). Obviously, type I variants are specific to the weeping pool, whereas type II and III variants are common to both pools. An important common character of the three informative segregation types is that the variant allele frequencies in the weeping pool are higher than those in the standard pool by 50 percentage points, providing a practicably measurable directional (positive) difference in variant allele frequency between the weeping and standard pools (Supplementary Table 1Supplementary Table S5; Fig. 3).

### Mapping of the weeping phenotype using weeping pool-specific variants

**Fig. 4.**
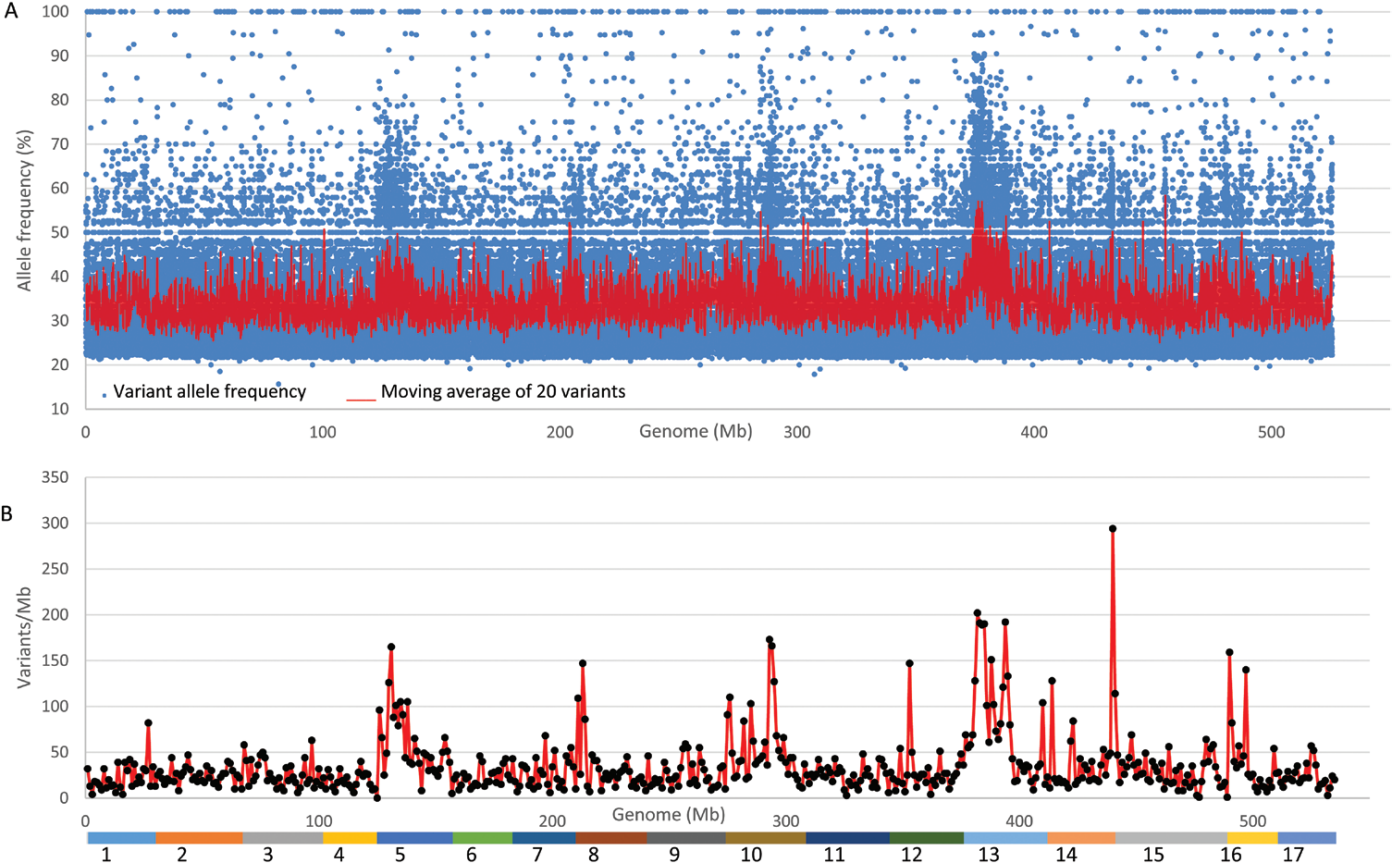
Distribution of allele frequency and density of variants specific to the weeping pool. (A) Distribution of allele frequency of 84562 variants. (B) Distribution of density of 18604 variants of allele frequency ranging from 40% to 60%. The colored bar at the bottom represents the assembled reference genome of 17 chromosomes as numbered. Based on *z*-score test, significant variant density peaks were detected on seven chromosomes, including 5 (*z*=4.0, *P*=6.4 × 10^−5^), 8 (*z*=3.4, *P*=6.7 × 10^−4^), 10 (*z*=4.2, *P*=2.6 × 10^−5^), 12 (*z*=3.4, *P*=6.7 × 10^−4^), 13 (*z*=5.1, *P*=3.4 × 10^−6^), 14 (*z*=7.9, *P*=0), and 16 (*z*=3.8, *P*=1.4 × 10^−4^).

Mapping of allele *W* was first conducted using the 84562 weeping pool-specific variants under the assumption that variants linked to the causal mutation for the weeping phenotype are heterozygous in ‘Cheal’s Weeping’ and homozygous in ‘Evereste’, i.e. following segregation type <**l**m×mm> (type I). Under this assumption, the average allele frequencies of these variants are anticipated to approach 50% in the *W* region (Supplementary Table 1Supplementary Table S5; Fig. 3C). To map allele *W*, the allele frequency data of the 84562 variants were plotted against the apple reference genome (Fig. 4A). To facilitate visual inspection, their average frequencies were calculated in a moving window of 20 variants. The results showed that the moving average frequency mostly ranged from 30% to 40% throughout the genome, consistent with their frequency distributions (Supplementary Table 1Supplementary Fig. S3A). Interestingly, there is a most visible region of a moving average allele frequency around 50%, as expected for type I variants, located on chromosome 13, suggesting that chromosome 13 putatively harbors the major locus *W*. In addition to chromosome 13, there appeared to be a number of regions on other chromosomes, such as 5 and 10, of allele frequencies around 50%, implicating uncertainties in allele frequency mapping.

To examine the uncertainties, the 18604 variants of allele frequency close to 50% (40–60%) were selected from the 84562 weeping pool-specific variants and were used to estimate variant density—the number of variants per million bp (Mb) DNA—throughout the genome (Fig. 4B). There were seven significant peaks of variant density, including the major peak on chromosome 13 (*z*=5.1, *P*=3.4 × 10^−6^) and others on chromosomes 5, 8, 10, 12, 14, and 16. Given the number of putative regions identified, we sought other approaches to confirm the findings.

Since weeping represents a naturally occurring mutation, this mapping strategy of using allele frequency of mutant pool-specific variants together with their variant density is dubbed mutant allele frequency and density (MAFD) mapping (Fig. 1viii).

### Mapping of the weeping phenotype using variants common to both pools

**Fig. 5.**
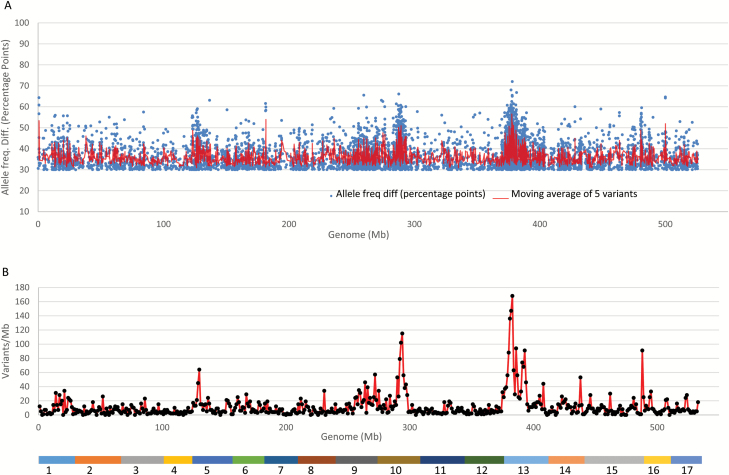
Distribution of allele frequency directional difference (AFDD) and density of variants common to both pools on the apple reference genome. (A) Distribution of AFDD of the 6377 variants of AFDD ≥30 percentage points between the weeping and standard pools. (B) Distribution of density of the 6377 variants. The colored bar at the bottom represents the assembled reference genome of 17 chromosomes as numbered. Significant variant density peaks were identified on chromosomes 13 (*z*=8.7, *P*=0), 10 (*z*=5.7, *P*=1.2 × 10^−8^), 16 (*z*=4.4, *P*=1.1 × 10^−5^), and 5 (*z*=2.9, *P*=3.7 × 10^−3^).

As shown earlier, variants common to both pools could be exploited for mapping based on type II and III variants. A positive 50 percentage point difference in allele frequency between the weeping and standard pools is expected for these two segregation types of variants. With this understanding, variants common to both pools of an allele frequency directional (positive) difference (AFDD) ≥30 percentage points would be sufficiently inclusive and informative to map allele *W*. In total, 6377 of the 173169 variants were found with an AFDD ≥30 percentage points. Plotting the 6377 variants against the genome revealed that there are seven regions on chromosomes 1, 5, 6, 10, 13, 15, and 16 that show a moving AFDD around 50 percentage points (Fig. 5A).

The density distribution of the 6377 variant of AFDD ≥30 percentage points in the genome indicated that there were four significant variant density peaks located on chromosomes 13 (*P*=0), 10 (*P*=1.2 × 10^−8^), 16 (*P*=1.1 × 10^−5^), and 5 (*P*=3.7 × 10^−3^) (Fig. 5B). Since they also were identified in MAFD (Fig. 4B) and AFDD (Fig. 5A) mappings, it was concluded that the four peak regions are significantly associated with weeping.

**Fig. 6.**
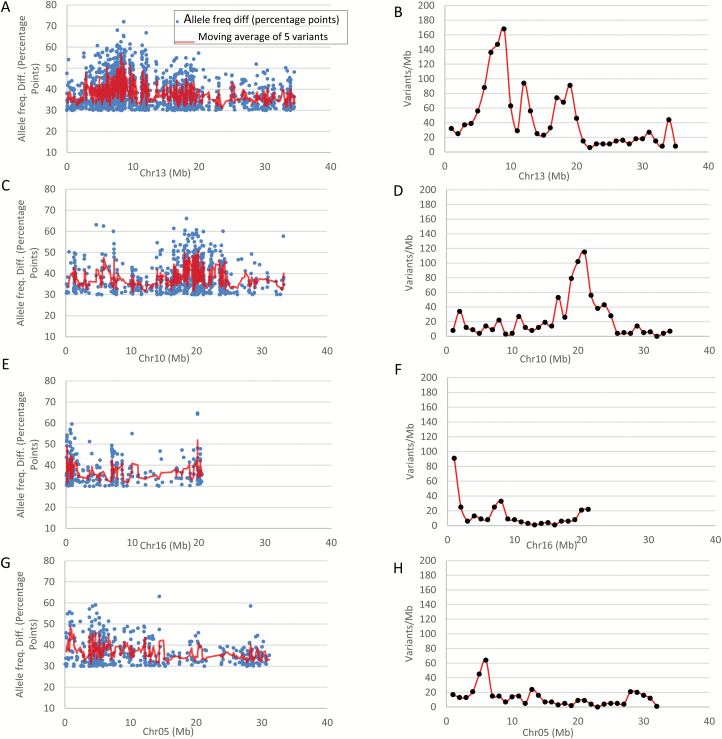
Distribution of allele frequency directional difference (AFDD) and density of variants with AFDD ≥30 percentage points on chromosomes 13 (A, B), 10 (C, D), 16 (E, F), and 5 (G, H).

A close look at the variant distribution for both AFDD and variant density revealed that the peaks covered: (i) a 7 (5th–12th)-Mb region on chromosome 13 (Fig. 6A, B), presumably including the weeping allele *W*; (ii) a 4 (18th–22nd)-Mb region on chromosome 10 (Fig. 6C, D), designated *W2*; (iii) the first 2-Mb region on chromosome 16 (Fig. 6E, F), designated *W3*; and (iv) a 3 (4th–7th)-Mb region on chromosome 5 (Fig. 6G, H), designated *W4*.

For convenience, such mapping processes that rely on DNA variants not only common to both pools but also with AFDD ≥30 percentage points and density polarity towards the pooling selection targeted genomic regions are called allele frequency directional difference and density (AFDDD) mapping (Fig. 1viii).

### Evaluation of variant genotype groups in the two pools targeted by AFDDD mapping

**Fig. 7.**
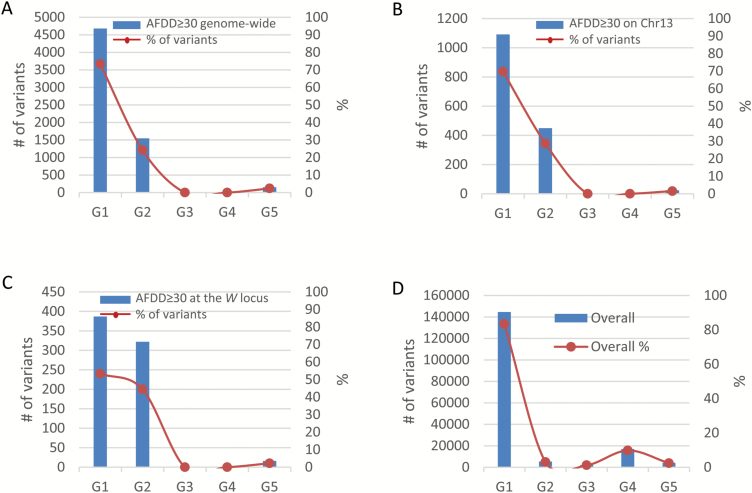
Assessing AFDDD mapping targeted variant genotype groups using the 6377 variants of AFDD ≥30 percentage points. (A–C) Number and frequency (%) of such variants observed in the five genotype groups at the genome scale (A), on chromosome 13 (B), and in the *W* region (C). (D) Number and frequency (%) of all the 173169 variants common to both pools in the five genotype groups. G1, heterozygous in weeping (He-W)/heterozygous in standard (He-S); G2, homozygous in weeping (Ho-W)/He-S; G3, He-W/homozygous in standard (Ho-S); G4, Ho-W/Ho-S; and G5, ‘Complex’. (This figure is available in color at *JXB* online.)

To see what and how the variant genotype groups in the two pools (Supplementary Table 1Supplementary Table S5; Fig. 3) were targeted in AFDDD mapping, their frequencies in the 6377 variants of AFDD ≥30 percentage points were analysed at the levels of genome, chromosome 13 and the 7-Mb region of *W* (Fig. 7A–C). Compared with the frequency of the 173169 variants common to both pools in the five genotype groups (Supplementary Table 1Supplementary Table S5; Fig. 7D), AFDDD mapping clearly selected for variants in G2, against G1, G3, and G4, and neutrally for G5. It drastically increased the frequency in G2 from 3.1% (Fig. 7D) to 24.3% at the genome level (Fig. 7A), to 28.7% on chromosome 13 (Fig. 7B), and to 44.4% in the *W* region (Fig. 7C). Meanwhile, AFDDD mapping decreased the frequency in G2 from 83.5% (Fig. 7D) to 73.4% (Fig. 7A), 69.8% (Fig. 7B), and 53.4% (Fig. 7C) at the three levels, respectively. Since groups G2 and G1 accounted for 97.8% in the *W* region, and variants in G2 are mostly, if not all, of segregation type <**l**m×**ll**> (type II), and only some of those in G1 are of genotype segregation type <**h**k×**h**k> (type III), AFDDD mapping primarily targets type II and type III variants (Supplementary Table 1Supplementary Table S5; Figs 3 and 7).

### Analysis of AFDDD mapping contributing segregation types

To examine the contributing roles of type II and type III variants to AFDDD mapping, two sets of variants were selected in the weeping pool from the 173169 variants common to both pools: one was the 15425 variants of allele frequency ≥95%; the other was the 12219 variants of allele frequency ranging from 70% to 80%. The assumptions are that under such selections in the weeping pool, the expected responses of type II and type III variants would show a marked increase in their numbers in the *w* region in the standard pool, which should be characterized with allele frequency close to 50% and 25%, respectively. A detailed analysis using these two sets of variants confirmed the assumptions (Supplementary Table 1Supplementary Figs S5 and Supplementary Table 1S6), highlighting their essential contributing roles in AFDDD mapping.

### DNA evidence in support of AFDDD mapping

**Fig. 8.**
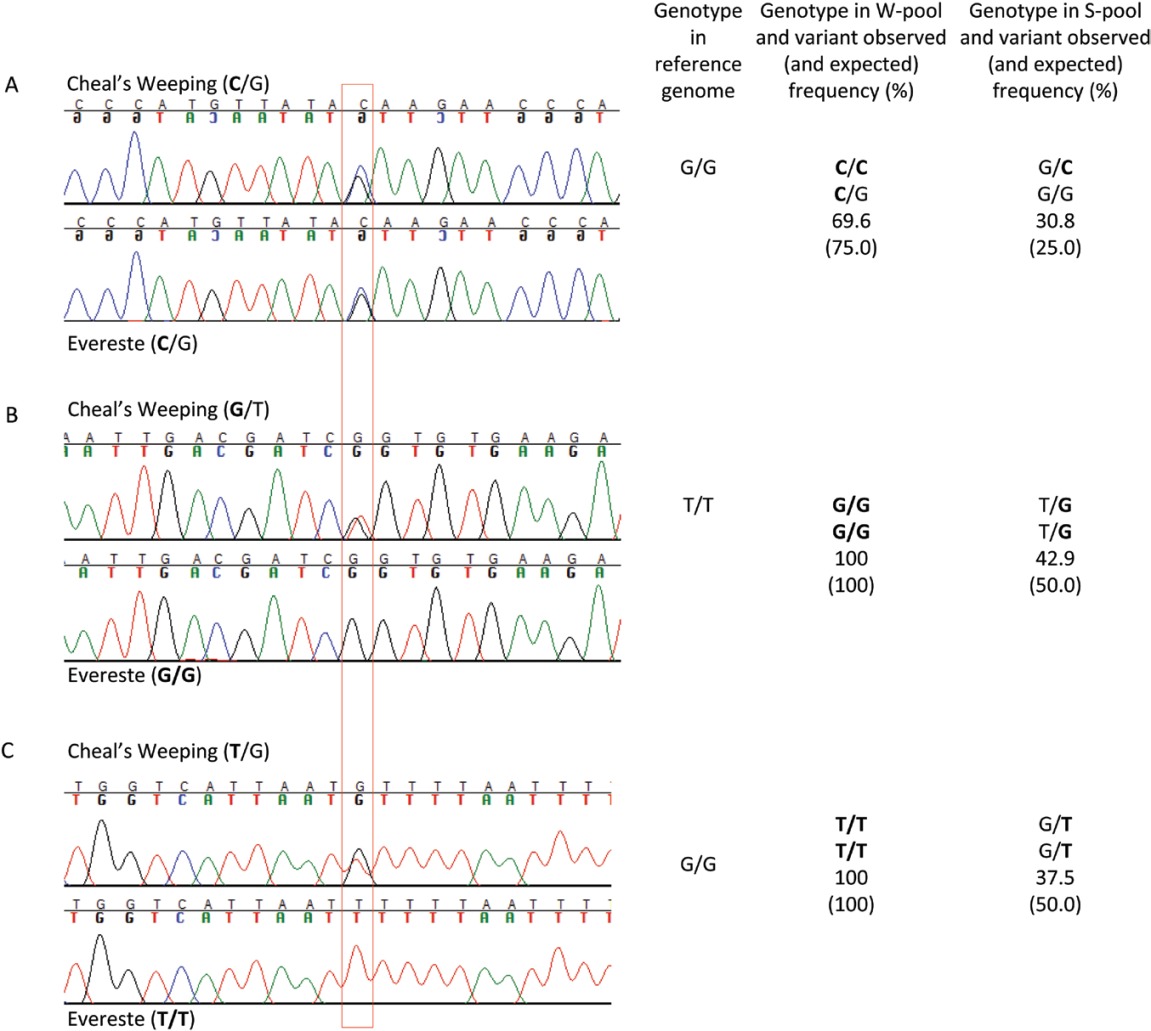
Chromatogram of DNA sequences of parents ‘Cheal’s Weeping’ and ‘Evereste’ covering three single nucleotide variants (SNVs; indicated by the red box) of segregation types <**h**k×**h**k> (A) and <**l**m×**ll**> (B, C) in the *W* region on chromosome 13. The SNV genotypes in the two parents, the reference genome, and the weeping and standard pools are listed accordingly. (A) SNV at position 7923460 in gene LOC103452418. (B) SNV at position 8209678 in gene LOC103452141. (C) SNV at position 8210175 in gene LOC103452141. Letters in bold represent DNA polymorphism (variant) in relation to the reference.

To directly confirm the presence of variants of segregation types <**l**m×**ll**> (type II) and <**h**k×**h**k> (type III), four genomic segments covering 14 putative variants (Supplementary Table 1Supplementary Table S3), including 12 for type II and two for type III in the *W* region, were PCR amplified from the two parents (‘Cheal’s Weeping’ and ‘Evereste’). Sanger DNA sequencing analysis of the PCR products confirmed that all the 14 variants had the expected genotypes in the two parents (Fig. 8; Supplementary Table 1Supplementary Table S3), providing physical evidence that variants of segregation types <**l**m×**ll**> and <**h**k×**h**k> are among those identified at the *W* locus by AFDDD mapping. Taken together, these data strongly support the role of type II and type III variants in AFDDD mapping.

### Confirmation of the mapping of locus W

**Fig. 9.**
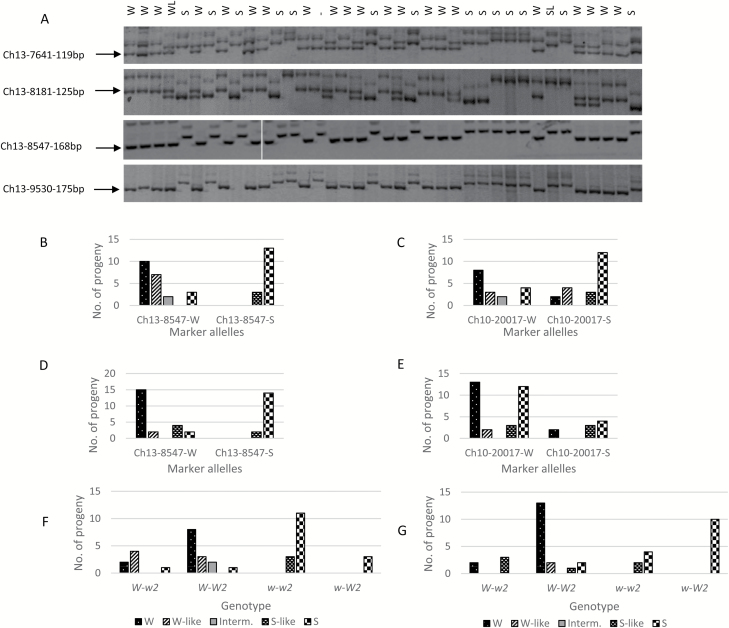
Confirmation of mapping of loci *W* and *W2.* (A) Analysis of four SSR markers Ch13-7641, Ch13-8181, Ch13-8547, and Ch13-9530 from the *W* region on chromosome 13 in population NY-051 × ‘Louisa’. The image shows the markers’ polyacrylamide gel electrophoresis profile in 38 of the 140 individuals. The SSR bands Ch13-7641-119bp, Ch13-8181-125bp, Ch13-8547-168bp (the vertical line between lanes 10 and 11 indicates that this marker was run in two gels), and Ch13-9530-175bp of ‘Louisa’ origin and linked to the weeping phenotype are indicated with an arrow. W, weeping; WL, weeping-like; S, standard; SL, standard-like; –, seedling tree was dead before phenotyping. (B–E) Weeping trait association of SSR markers Ch13-8547 (in the *W* region) and Ch10-20017 (in the *W2* region) in populations ‘Cheal’s Weeping’ × ‘Evereste’ (B, C) and NY-011 × NY-100 (D, E). Marker alleles linked to weeping and standard are suffixed with ‘-W’ and ‘-S’, respectively. (E, F) Effect of genetic interactions between the alleles of *W* and those of *W2* (deduced from marker alleles Ch13-8547-W and Ch10-20017-W, respectively) on the expressivity of the weeping phenotype in populations ‘Cheal’s Weeping’ × ‘Evereste’ (E) and NY-011 × NY-100 (F).

To confirm the mapping of *W*, four SSR markers from the 7-Mb region of *W* (Supplementary Table 1Supplementary Table S3) were developed and analysed. In population NY-051 × ‘Louisa’, based on the genotypic data and the progeny growth habit evaluations (Figs 2B and 9A), markers SSR7641 and SSR8181 flank the *W* locus by three and one recombinants, respectively, from one side, and marker SSR9530 flanks *W* from the other side by two recombinants, delimiting the *W* locus within a 1.4 (8.1th–9.5th)-Mb genomic region. Marker Ch13-8547 co-segregates with the weeping phenotype (intermediates discounted), confirming the mapping of *W*.

In population ‘Cheal’s Weeping’ × ‘Evereste’, marker Ch13-8547 segregated 17:21 for the *w*- and *W*-linked alleles, respectively, from ‘Cheal’s Weeping’ (Fig. 9B). Of the 17 progeny of the *w*-linked-allele, 14 were scored standard and three were standard-like, demonstrating a complete linkage to *w*. However, the 21 individuals of the *W*-linked allele were observed with a range of scores, including weeping (10), weeping-like (7), intermediate (2), and standard (2) (Fig. 9B).

In population NY-011 × NY-100, similar results were observed. Marker Ch13-8547 segregated with 16:23 for the *w*- and *W*-linked-alleles, respectively (Fig. 9D). The 16 progeny carrying the *w*-linked-allele showed normal growth habit, including 14 standard and two standard-like. Among the 23 individuals of the *W*-linked allele, 15 were noted as weeping, two as weeping-like, four as standard-like and two as standard (Fig. 9D). Therefore, these data confirmed the mapping of the major locus *W* on chromosome 13 in the three populations although the locus *W* could not explain the observations that eight progeny carrying the *W*-allele showed standard or standard-like phenotypes in populations ‘Cheal’s Weeping’ × ‘Evereste’ and NY-011 × NY-100 (Fig. 9B, D).

### Confirmation of the mapping of locus W2

For confirmation of the mapping of *W2*, three SSR markers (Supplementary Table 1Supplementary Table S3) from the *W2* region were developed and evaluated. In population NY-051 × ‘Louisa’, markers Ch10-19768 and Ch10-20017 segregated normally, but did not show significant association with the weeping phenotype (data not shown), suggesting that mapping of *W2* could not be confirmed.

In population ‘Cheal’s Weeping’ × ‘Evereste’, the segregation of markers Ch10-19768 and Ch10-20017 showed a significant association with the weeping phenotype. For example, marker Ch10-20017 segregated the *w*-linked allele in 21 individuals and the *W*-linked allele in 17, fitting the 1:1 ratio (χ^2^=0.237, *P*=0.626) as expected (Fig. 9C). Among the 21 progeny of the *w*-linked allele, the ratio between weeping (-like) and standard (-like) was 6:15, significantly skewed towards the standard phenotype from the expected 1:1 ratio (χ^2^=3.86, *P*=0.049). In contrast, a considerable skewness towards the weeping phenotype in the 17 progeny carrying the *W*-linked allele was observed (χ^2^=3.27, *P*=0.071) as there were 11 weeping (-like) and only four standard (-like) individuals (Fig. 9C). The remaining two were intermediate and were not counted.

In population NY-011 × NY-100, markers Ch10-20017 and Ch10-20761 (Supplementary Table 1Supplementary Table S3) were informative for the weeping associated allele from ‘Red Jade’. Unlike what was observed in population ‘Cheal’s Weeping’ × ‘Evereste’, the segregation of marker Ch10-20017 was significantly distorted from 1:1 (χ^2^=10.26, *P*=1.36 × 10^–3^) as its *W*- and *w*-linked alleles were observed in 30 and 9 progeny, respectively (Fig. 9E). A close look revealed that the marker segregated 15:7 for the *W*- and *w*-linked alleles in the 22 progeny of standard (-like) phenotype, fitting the ratio 1:1 (χ^2^=2.227, *P*=0.136). However, the marker segregated 15:2 for in the 17 progeny of weeping (-like) phenotype, significantly skewed towards weeping (χ^2^=8.471, *P*=3.60 × 10^−3^), suggesting a significant linkage between marker Ch10-20017 and the weeping phenotype, confirming again the mapping of *W2*.

### Genetic interactions between *W* and *W2*

Investigating the genetic interactions between the *W* and *W2* alleles indicated that allele *W* was required for the weeping phenotype as there were no weeping individuals scored when it was absent in genotypes *w–w2* and *w–W2* (Fig. 9F, G) in the two populations. Comparing the number of individuals of typical weeping phenotype in genotype *W–W2* with that in genotype *W–w2*, i.e. 8/14 (57.1%) *vs* 2/7 (28.6%) in ‘Cheal’s Weeping’ × ‘Evereste’, and 13/18 (72.2%) *vs* 2/5 (40.0%) in NY-011 × NY-100, suggested that the presence of allele *W2* increased the penetrance of the weeping phenotype from allele *W* (Fig. 9F, G). Notably, one of the two *W*-carrying progeny of standard phenotype was genotyped as *W–w2* in ‘Cheal’s Weeping’ × ‘Evereste’, providing a likely cause for the observation although the other remains to be explained due to its genotype *W–W2*. The *W–w2* genotype appeared to be also responsible for the observation that there were three *W*-carrying progeny of standard-like phenotype in population NY-011 × NY-100 (Fig. 9G).

### Identification of differentially expressed genes in the *W* and *W2* regions

Based on the first version (V1) of the apple reference genome (Velasco *et al.*, 2010; Bai *et al.*, 2014), there are 153 and 368 genes or transcribed sequences in the *W* (1.4 Mb) and *W2* (4 Mb) regions. To examine their expression patterns, an RNA-seq analysis was performed using actively growing shoot tip tissues from four pooled weeping and four pooled standard progeny in ‘Cheals Weeping’ × ‘Evereste’. A total of 43.2 million raw reads were obtained for the weeping pool and 59.2 million for the standard pool (Supplementary Table 1Supplementary Table S6, NCBI accession SRP094968). The RNA-seq analysis, which was validated by qRT-PCR testing on ten selected genes (Supplementary Table 1Supplementary Fig. S7), identified 79 genes expressed (RPKM ≥1.0 in at least one of the RNA-seq pools) in the *W* region (Supplementary Table 1Supplementary Table S7) and 199 in the *W2* region (Supplementary Table 1Supplementary Table S8). There are three differentially expressed genes (DEGs) (*P*_FDR_<0.05) in the *W* region, including MDP0000928608 (M928608) and M534197 (both glucuronoxylan 4-*O*-methyltransferase (GXMT)-like genes), and M160372 (a rubber elongation factor-like gene). In the *W2* region, five genes were expressed differentially, including G103289 and G104254 (both TMV resistance protein N-like genes), G102554 (an AUX/IAA7.1-like gene), M142356 (a chloroplastic decapping nuclease DXO-like gene), and M819881 (an E3 ubiquitin-protein ligase 3-like gene), where ‘G######’ represents novel transcripts (Bai *et al.*, 2014).

In the latest version (V2) of the apple reference genome (Daccord *et al.*, 2017), the *W* and *W2–W4* regions were all found on their corresponding chromosomes as those in V1 (Velasco *et al.*, 2010; Bai *et al.*, 2014) according to a BLAST-based dot matrix analysis although their chromosomal nucleotide coordinates were different (Supplementary Table 1Supplementary Fig. S8A–D). The *W* region between markers SSR8181 and SSR9530 was found in a less than 1-Mb segment from the 8.6th to the 9.6th Mb on chromosome 13 (Supplementary Table 1Supplementary Fig. S8A) that contains 72 predicted genes, whereas the *W2* region was determined to span over 3.5 Mb from the 27.5th to the 31.0th Mb on chromosome 10 (Supplementary Table 1Supplementary Fig. S8B), where 216 genes are annotated. RNA-seq analysis (Supplementary Table 1Supplementary Table S6) showed that 60 of the 72 genes in the *W* region (Supplementary Table 1Supplementary Table S9) were expressed, of which eight were DEGs, including three (MD13G1118800, MD13G1119900, and MD13G1120100) that correspond to the three DEGs identified in V1 (Supplementary Table 1Supplementary Tables S7, Supplementary Table 1S9, and Supplementary Table 1S10). The remaining five DEGs include three (MD13G1119100, photosystem I light harvesting complex gene 2; MD13G1121000, nuclear transport factor 2A; and MD13G11233000, BCL-2-associated athanogene 5) that were non-DEGs in V1 and two (MD13G11271000, jasmonate-zim-domain protein 10; and MD13G1127300, acyl-CoA oxidase 1) that were immediately outside the *W* region in V1 (Supplementary Table 1Supplementary Table S10). In the *W2* region, 151 of the 216 genes were expressed and six of them were DEGs (Supplementary Table 1Supplementary Table S11). Three (MD10G1192900, MD10G1199900, and MD10G1202900) of the six DEGs were identified in V1 as well, equivalent to G104254, M142356, and M819881, respectively (Supplementary Table 1Supplementary Table S10). The other three DEGs include MD10G1196900 (glutathione *S*-transferase TAU 19) and MD10G1203300 (ribosomal protein S5/elongation factor G/III/V family protein) that were non-DEGs in V1, and MD10G1196600 (glutathione *S*-transferase TAU 25) that was not annotated in V1.

## Discussion

### Challenges in pooled genome sequencing-based genetic mapping in *Malus* and MAFD and AFDDD mappings

DNA variants of segregation type <**l**m×mm> (type I) in the weeping (mutant) pool are an obvious target to be exploited in genetic mapping studies involving an F_1_ population derived from two parents of heterogeneously heterozygous genome such as ‘Cheal’s Weeping’ × ‘Evereste’. We anticipated that application of MAFD mapping using the weeping pool-specific variants would lead to a relatively ‘clean’ mapping of the weeping phenotype as it uses an approach analogous to a combination of MAF mapping (Schneeberger, 2014) and variant density estimates similar to what was described previously (Zuryn *et al.*, 2010; Minevich *et al.*, 2012). This was true for mapping the major weeping locus *W*, but the allele frequency-based approach was found to have generated a number of other regions putatively associated with the weeping phenotype (Fig. 4A). The variant density-based approach appeared to provide improved certainty, but it reported the *W* locus along with six other genomic regions significantly associated with weeping (Fig. 4B), raising questions about the utility of such a straightforward adaptation of existing mapping strategies to the most commonly used type I variants.

AFDDD mapping is an approach developed to address the uncertainty issue encountered in MAFD mapping by unlocking genetic information from DNA variants of other hidden segregation types for mapping. It differs from MAFD mapping in the follow ways: (i) AFDDD mapping focuses on variants of segregation types <**l**m×**ll**> (type II) and <**h**k×**h**k> (type III) rather than <**l**m×mm> (type I); (ii) it uses the common variants between the pools rather than the weeping pool-specific variants; and (iii) it examines variant allele frequency directional differences (AFDD) between the weeping and standard pools rather than their original allele frequency. Similar to MAFD mapping but distinct from other approaches, such as delta SNP index mapping (Fekih *et al.*, 2013; Takagi *et al.*, 2013), AFDDD mapping also emphasizes variant density by examining how variants were distributed preferentially in the pooling selection targeted regions rather than in the other non-linked regions. As such, AFDDD mapping was found to be an effective approach for identifying regions of major or minor effect, similar to QTLs, on weeping in *Malus* (Figs 5 and 6).

An advantage in AFDDD mapping is that the variants common to both pools are of higher accuracy than the pool-specific variants. This is likely due to the fact that common variants were each recognized twice, once in the weeping pool and again in the standard pool, whereas the pool-specific variants were called only once in one of the two pools. It is common to see genomic regions of uneven sequencing coverage between the pools. If a variant is called from a region in one pool, but not called in the same region in the other pool due to lower coverage, the variant would be falsely identified as specific to the first pool. In search of genetic variations between apple cultivars ‘Gala’ and ‘Blondee’, a yellow fruit somatic mutation of ‘Gala’ using RNA-seq data, ‘Blondee’-specific variants were proven to be false positives due to uneven coverage (El-Sharkawy *et al.*, 2015). Since the pool-common variants must be called in both pools, such false positive variants can be eliminated. In addition, DNA variant calling has been challenging in NGS data analysis due to false positive issues (Huang *et al.*, 2015; Ribeiro *et al.*, 2015).

A drawback in AFDDD mapping is that the causal mutations cannot be readily identified using the variants common to both pools for a dominant trait. This drawback of AFDDD mapping, however, underscores a major advantage in MAFD mapping as the causal mutations would be present in the mutant pool-specific variants. Therefore, for improving the mapping results and for identifying candidate causal mutations, simultaneously using AFDDD mapping together with MAFD mapping provides an improved strategy for pooled genome sequencing data analysis in *Malus*. An alternative approach to address the drawback in AFDDD mapping is to conduct another round of AFDDD mapping by copying a small fraction of randomly selected reads in the mutant pool, e.g. equivalent to 10–15% of reads in the wild type pool, into the wild type pool before variant calling. It is expected that such manipulation in read pooling would make the causal variants detectable in the modified wild type pool as long as a lower variant read count threshold (e.g. 10–15%) is used in variant calling.

Allelic distance (AD) is defined as the sum of absolute differences in the allele (variant) counts within a given region between two pools. AD mapping has been shown to be useful for mapping mutation using pooled genome sequencing data (Schneeberger, 2014). The genetic presumptions are that the allelic distance between two pools of contrasting phenotype would be the largest in the causal region as the alleles of the mutant background are expected to be more concentrated there than in other non-linked regions (Schneeberger, 2014). Since AD mapping relies on counting the number of variants, including pool-specific and pool-common variants, this likely would make it less effective in heterogeneously heterozygous species. In this study, if AD mapping were used, the input variants would include those both specific and common to the pools, i.e. 349859 (sum of 84562 weeping pool-specific, 92148 standard pool-specific, and 173169 common to both pools; Fig. 1vi). To be effective, the 173169 common variants would be excluded to avoid the results being obscured. This would virtually be equivalent to conducting the variant density assays between the two pools. Since the *w* region on chromosome 13 also showed a significantly higher variant density (data not shown), using AD mapping would have rendered the major locus *W* undetectable.

### Genetic basis of AFDDD mapping

Through a detailed analysis of allele frequency and genotype of variants in the weeping and standard pools, a series of hypothetical variant segregation types were inferred, leading to identification of three that were used for mapping, including <**l**m×mm> suitable for the weeping pool-specific variants, and <**h**k×**h**k> and <**l**m×**ll**> for variants common to both pools (Supplementary Table 1Supplementary Table S5; Fig. 3A–C). Although DNA variants of segregation type <**l**m×mm> (type I) are commonly used, variants of segregation types <**l**m×**ll**> (type II) and <**h**k×**h**k> (type III) have not been employed in pooled genome sequencing studies in out-crossing woody species. Successful mapping of loci *W*, *W2*, *W3*, and *W4* based on type II and type III variants, and the DNA sequence confirmation of 14 such variants in both weeping and non-weeping parents ‘Cheal’s Weeping’ and ‘Evereste’ (Fig. 8) suggested that they represent a unique source of DNA variants that can be exploited in NGS-based pooled genome sequencing analysis. Indeed, among the 173169 variants common to both pools, 5353 (3.1%) are type II variants, and type III would be even more (Supplementary Table 1Supplementary Table S5; Fig. 7). In addition, this study has also demonstrated such variants are present throughout the genome and were readily identifiable and selectable through genome pooling (Fig. 7; Supplementary Table 1Supplementary Figs S5 and Supplementary Table 1S6).

Comparing allele frequencies is necessary in all studies involving analysis of pooled DNA samples (Shaw *et al.*, 1998). In classic BSA (Giovannoni *et al.*, 1991; Michelmore *et al.*, 1991), identification of markers linked to a trait of interest is accomplished by analysing the marker allele frequency difference between two groups of individuals forming the two pools and a level of difference at 100% is typically pursued (Xu *et al.*, 2000). In pooled genome sequencing-based mapping studies, variant allele frequency differences have also been exploited, such as delta SNP index mapping (Fekih *et al.*, 2013; Takagi *et al.*, 2013). In AFDDD mapping, the optimal AFDD level is 50 percentage points between the weeping and standard pools as determined by type II and type III variants. In this study, the threshold of AFDD≥30 percentage points was chosen to accommodate deviations. Such a magnitude of AFDD is likely the underlying reason for a ‘clean’ mapping of the weeping phenotype using variant density, including the elimination of the highest density peak on chromosome 14 identified using type I variants (Fig. 4B).

Variant allele density is likely a parameter more important in AFDDD mapping than in MAFD as it performed better for mapping the weeping phenotype. Due to the inherent selection during genome pooling and local physical linkage, the causal mutation region will inevitably have a higher density of variants than in the unlinked region in the pools (Schneeberger *et al.*, 2009; Zuryn *et al.*, 2010; Minevich *et al.*, 2012), explaining the effectiveness of variant density as a mapping parameter. Interestingly, a recent study (Jensen *et al.*, 2014) in an apple rootstock segregation population reported that most of the DEGs associated with resistance to powdery mildew and woolly apple aphid were clustered in a 9–10 Mb region. However, the underlying genetic basis for such observations likely differs from that for the MAFD and AFDDD mappings.

### Genetic control of weeping in *Malus*

The weeping phenotype in other woody species is controlled mostly by a single recessive allele, such as *pl* (Dirlewanger and Bodo, 1994) or *we* (Chaparro *et al.*, 1994) in peach (*Prunus persica*), *pl* (Zhang *et al.*, 2015) in mei or Japanese apricot (*Prunus mume*), and *wp1* (Roberts *et al.*, 2015) in eastern redbud (*Cercis canadensis*). In the case of mei, some modifier genes were likely involved in how the weeping phenotype is expressed in addition to *pl* (Zhang *et al.*, 2015). Non-allelic weeping alleles were reported as well. For example, the weeping allele *wp1* is non-allelic to another weeping phenotype in eastern redbud (Roberts *et al.*, 2015).

Using NGS-based MAFD and AFDDD mapping approaches, the present report uncovered four chromosomal regions, *W* (chr13), *W2* (chr10), *W3* (chr16), and *W4* (chr5) (Fig. 6), that are significantly associated with the weeping phenotype inherited from ‘Cheal’s Weeping’. This appeared to be consistent with what was suggested in another study using ‘Red Jade’ as a weeping parent (Just, 2001), where a number of progeny of intermediate phenotype were documented. Together with the independent confirmation of loci *W* and *W2* and the investigation into their genetic interactions (Fig. 9), these findings provided important insight into the genetic control of weeping in *Malus*. The *W* locus is clearly of the most significant influence on weeping. For this reason, it is regarded as the same locus for the major dominant gene *W* previously reported for *M. baccata* ‘Gracilis’ (Sampson and Cambron, 1965; Brown, 1992; Alston *et al.*, 2000). Allele *W2* has been shown to play an important role in the expressivity of the weeping trait under the background of allele *W* in populations ‘Cheal’s Weeping’ × ‘Evereste’ and NY-011 × NY-100, but a non-detectable role in population NY-051 × ‘Louisa’. This indicates that ‘Louisa’ likely differs from ‘Cheal’s Weeping’ and ‘Red Jade’ in the genetic mechanism responsible for the expressivity of the weeping phenotype, suggesting that ‘Louisa’ is distinct while ‘Cheal’s Weeping’ and ‘Red Jade’ are related each other.

A study of the relatedness in a diverse set of *Malus* weeping accessions, based on genotypic data from seven SSR markers, showed that weeping accessions in crabapple were clustered into two clades: one is the *M. prunifolia* ‘Pendula’ and its descendants, and the other is the ‘Hyvingiensis’ group (Lindén and Iwarsson, 2014). The study also reported that apple cultivar ‘Elise Rathke’ accessions formed their own group distinct from all weeping accessions in crabapples. An important future study would be to understand if the *W* locus and any of the three other loci, *W2*, *W3*, and *W4*, are also the key genetic factors determining their weeping phenotype.

Identification of the causal mutation is an important goal in pooled genome sequencing studies. Successful identification of causal mutations has been documented (Schierenbeck *et al.*, 2015; Huo *et al.*, 2016; Petit *et al.*, 2016). In this study, three or eight genes in the *W* region and five or six genes in the *W2* region were expressed differentially depending on the versions of the apple reference gnome used (Supplementary Table 1Supplementary Tables S7–S11). In the *W* region, the two GXMT-like genes, M534197 (MD13G1120100) and M160372 (MD13G1119900), are of more interest. It is known that GXMT-like genes function in biosynthesis of the hemicellulose 4-*O*-methyl glucuronoxylan, a major component of the secondary cell walls in dicots (Urbanowicz *et al.*, 2012), and mutations in xylan synthesis genes often lead to unusual growth of plants (Carpita and McCann, 2015). Among the five or six DEGs in the *W2* region, the *AUX/IAA7.1*-like gene G102554 (MD10G1192900) appeared to fit well the role of *W2*. Several characterized *AUX/IAA* like genes in Arabidopsis were shown to act as a repressor of auxin-inducible gene expression and to play roles in the control of gravitropic growth and development in light-grown seedlings (Yu *et al.*, 2013; Sato *et al.*, 2014, 2015).

The other genes of putatively regulatory roles in plant growth but not expressed differentially in statistics between weeping and standard progeny may not be ruled out. In the *W* region, these genes, for example, may include a LAZY1-like gene, M254069 (MD13G1122400), and a transcription factor TCP20-like gene, M374900 (MD13G1122900), as LAZY1s are involved in plant response to gravitropism (Li *et al.*, 2007; Yoshihara and Iino, 2007; Yoshihara *et al.*, 2013) and TCP20s regulate plant basic cellular growth process (Johnson and Lenhard, 2011; Guan *et al.*, 2014) (Supplementary Table 1Supplementary Tables S7, Supplementary Table 1 S9, and Supplementary Table 1 S10). Similarly, in the *W2* region, the *ATAUX2-11*-like gene M176753 (MD10G1193000) and the small auxin-up RNA (SAUR)-like genes M186167 (MD10G1202100) and M138076 (MD10G1204800) also appeared to be interesting candidates (Supplementary Table 1Supplementary Tables S8, Supplementary Table 1 S10, and Supplementary Table 1 S11). Several studies reported that *ATAUX2-11* is an auxin and gravitropic responsive transcription factor (Wyatt *et al.*, 1993; Riechmann *et al.*, 2000) and SAUR proteins are key regulators for plant growth and development (Ren and Gray, 2015), including plant branch angle (Bemer *et al.*, 2017). Overall, the findings reported here represent an important step forward to a more comprehensive understanding of the weeping phenotype in *Malus*. However, due to the complex genome in *Malus*, a dedicated effort is required for revealing the identity of alleles *W* and *W2* and possibly *W3* and *W4*.

## Conclusions

In an F_1_ population developed in out-crossing woody species, there are at least three segregation types of DNA variants that are informative for genetic mapping using pooled genome sequencing analysis: <**l**m×mm> (type I) in the mutant (weeping) pool-specific variants and <**l**m×**ll**> (type II) and <**h**k×**h**k> (type III) in the variants common to both mutant and wild type pools. Type I variants are commonly the first choice for mapping, and they are expected to include causal variants. Mapping using type I variants could be readily performed through MAFD mapping, but false positives may reduce the efficacy of this approach. Type II and type III variants are important and a more effective alternative for mapping, but causal variants are unlikely to be covered for dominant traits. AFDDD mapping is an effective approach to target type II and type III variants. Variant density appeared to be a better parameter than variant allele frequency in mapping. Both MAFD and AFDDD mappings can be applied for QTL discovery. There are four genomic regions of significant association with the weeping phenotype in *Malus*, including a major locus *Weeping* (*W*) on chromosome 13 and three others on chromosomes 10 (*W2*), 16 (*W3*), and 5 (*W4*). Confirmation of the mapping of *W* and *W2*, investigation into their genetic interactions, and identification of expressed genes in the *W* and *W2* regions shed light on the genetic control of the weeping trait and its expressivity in *Malus*.

## Supplementary data

Supplementary data are available at JXB online.

Fig. S1. A typical weeping and a standard F_1_ progeny from population ‘Cheal’s Weeping’ × ‘Evereste’.

Fig. S2. Genotype frequency of DNA variants specific to the weeping and standard pools and common to both weeping and standard pools from population ‘Cheal’s Weeping’ × ‘Evereste’.

Fig. S3. Distribution of pool-specific and common variants.

Fig. S4. Schematics for possible segregation types inferred for variant genotype group G5 ‘Complex’.

Fig. S5. Evaluating the role of segregation type <**l**m×**ll**> (type II variants) in AFDDD mapping using common variants (15425) selected of allele frequency ≥95% in the weeping pool.

Fig. S6. Evaluating the role of segregation type <**h**k×**h**k> (type III variants) in AFDDD mapping using common variants (12219) selected of allele frequency ranging from 70% to 80% in the weeping pool.

Fig. S7. qRT-PCR validation of gene expression in RNA-seq analysis.

Fig. S8. BLAST-based dot matrix analysis of the genomic regions associated with the weeping trait between two versions of the apple reference genome.

Table S1. Reads mapping summary.

Table S2. Variant filtering process.

Table S3. Primer sequences and their genome physical locations.

Table S4. qRT-PCR primer sequences and their targeted gene IDs.

Table S5. Genotype groups of variants common to both pools and variant segregation types inferred.

Table S6. Summary of RNA-seq reads mapping.

Table S7. List of expressed genes in the *W* region.

Table S8. List of expressed genes in the *W2* region.

Table S9. List of expressed genes in the *W* region according to the new reference genome (Daccord *et al.* 2017).

Table S10. Differentially expressed genes (DEG) and genes of interest in the *W* and *W2* regions according to both versions of the apple reference genome.

Table S11. List of expressed genes in the *W2* region according to the new reference genome (Daccord *et al.* 2017).

supplementary_figures_S1_S8_Tables_S1_S6

supplementary_tables_S7_S11

## Abbreviations:

AFD: allele frequency difference
AFDD: allele frequency directional difference
AFDDD: AFDD and density
BSA: bulked segregant analysis
DEG: differentially expressed gene
MAFD: mutant allele frequency and density
NGS: next generation sequencing
SNV: single nucleotide variant;
QTL: quantitative trait locus.
