# Supplementary material for: Exploring DNA variant segregation types in pooled genome sequencing enables effective mapping of weeping trait in Malus
Source: J Exp Bot. 2018 Jan 29;69(7):1499–516. doi: 10.1093/jxb/erx490 (PMC5888915; doi:10.1093/jxb/erx490)
Supplement: supplementary_figures_S1_S8_Tables_S1_S6 [file erx490_suppl_supplementary_figures_s1_s8_tables_s1_s6.pdf]

Supplementary Information

Exploring DNA variant segregation types in pooled genome sequencing enables effective mapping of weeping trait in *Malus*

Short Running Title:

Laura Dougherty[1,3], Raksha Singh[1,3], Susan Brown[1], Chris Dardick[2] and Kenong Xu[1*]

[1]Horticulture Section, School of Integrative Plant Science, Cornell University, NYSAES, Geneva, NY 14456, USA.

[2]USDA-ARS Appalachian Fruit Research Station, Kearneysville, WV 25430, USA.

[3]These authors contributed equally to this work

***Corresponding author**: Kenong Xu

Address: Horticulture Section, School of Integrative Plant Science, Cornell University, New York State Agricultural Experiment Station, Geneva, NY 14456, USA

Telephone: .315-787-2496

Fax: 315-787-2216

E-mail: kx27@cornell.edu

Supplementary Figures: 8

Supplementary Tables: 11

Supplementary Figure S1

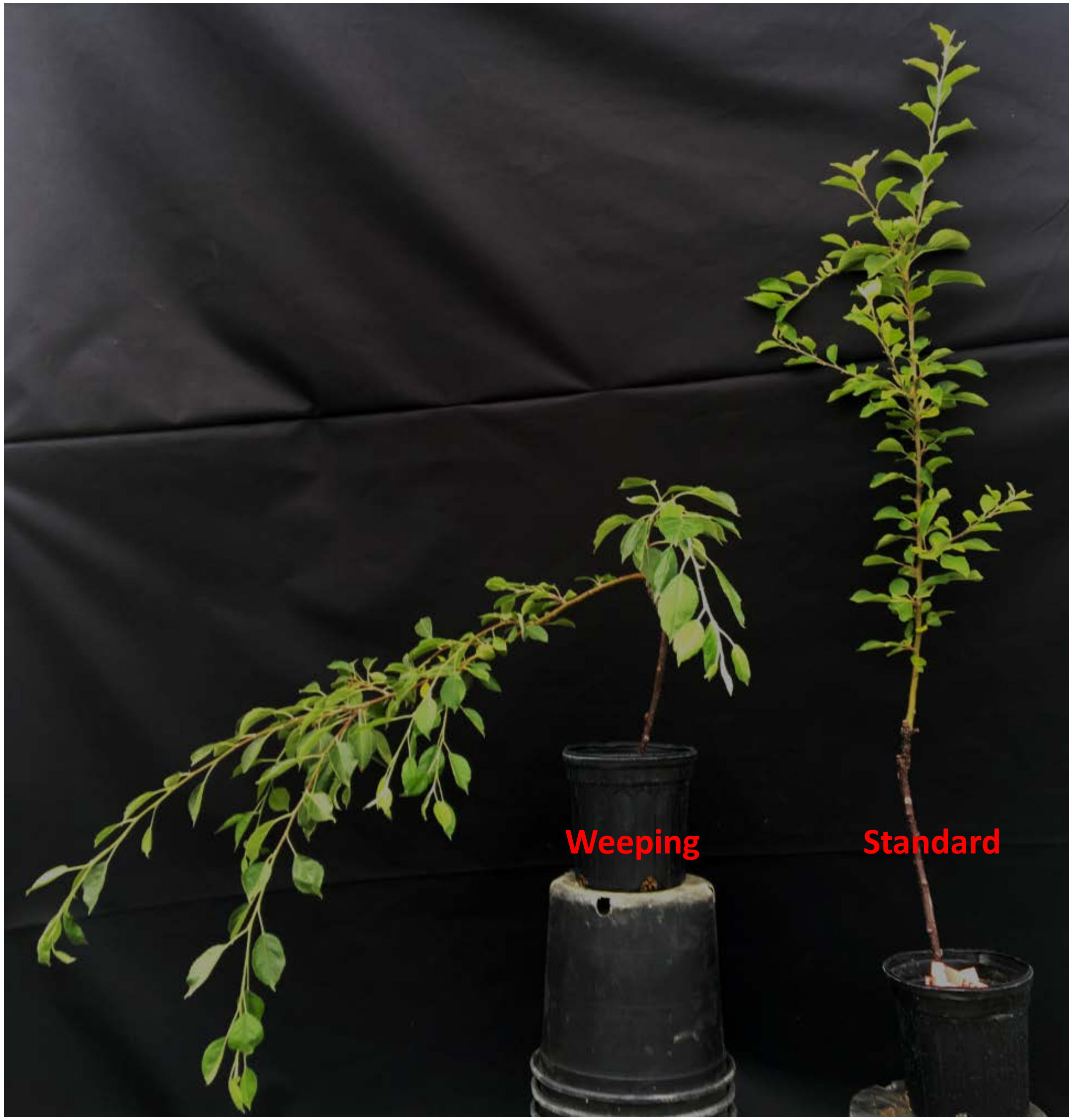

Fig. S1. A typical weeping and a standard F_1 progeny from population ‘Cheal’s Weeping’ × ‘Evereste’ after being budded for 1.5 years on apple rootstock B118.

Supplementary Figure S2

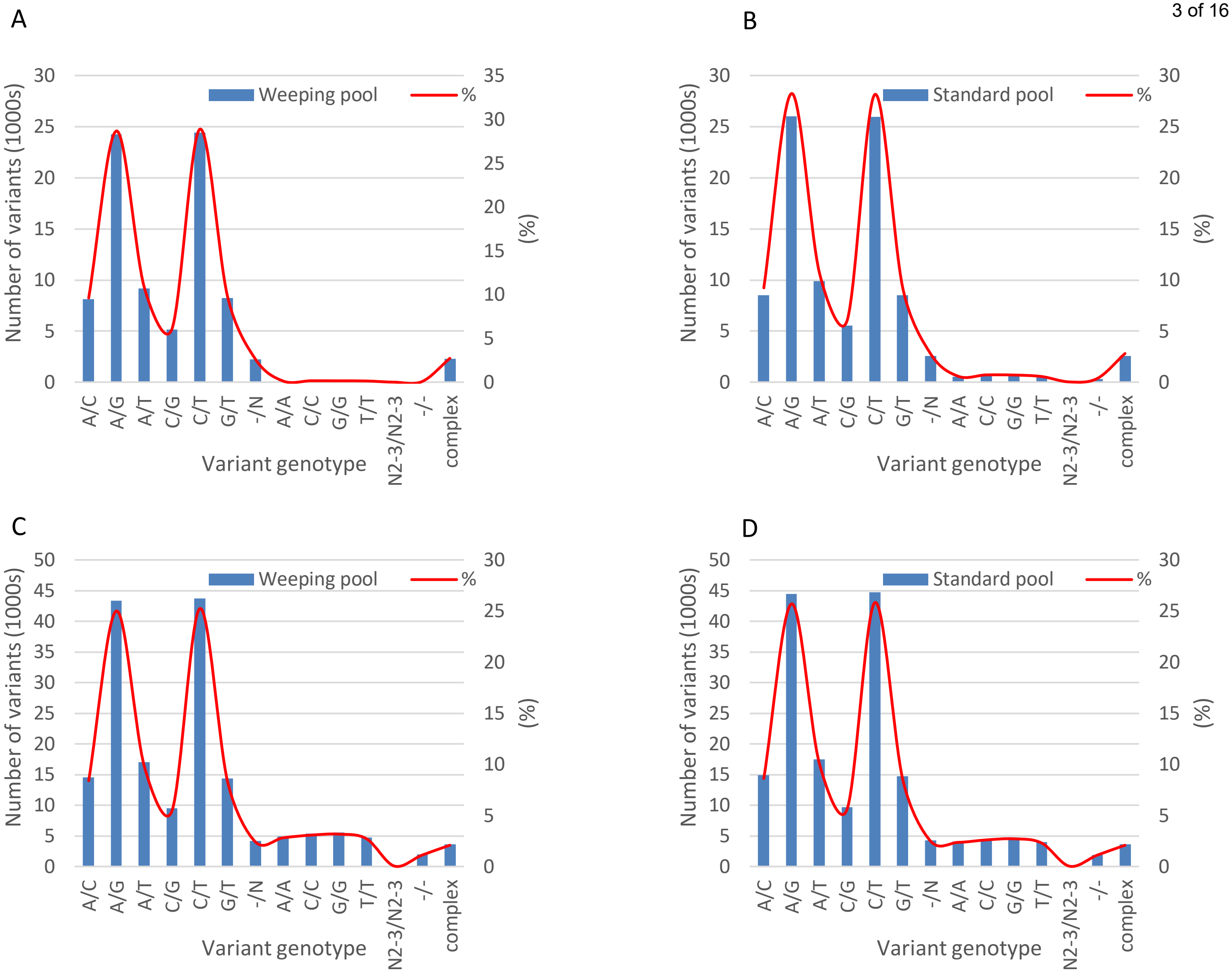

Fig. S2. Genotype frequency of DNA variants specific to the weeping (A) and standard (B) pools and common to both weeping (C) and standard (D) pools from population 'Cheal's Weeping' x 'Evereste'. 'N2-3' and '-'stand for 2- or 3-nucleotide variants and InDels, respectively. In the pool specific variants, heterozygous variants comprising seven groups A/C, A/G, A/T, C/G, C/T, G/T, and -/N (heterozygous InDel) were predominant, accounting for 96.6% in weeping and 94.4% in standard pools (A, B). Variant genotypes A/G and C/T were most common and each explained 28.2-28.9%. In the variants common to both pools, a similar trend was observed. However, heterozygous variants were lower, denoting 84.8% in weeping and 86.8% in standard while homozygous variants, mainly A/A, C/C, G/G and T/T, accounted for 13.1% and 11.1% in weeping and standard pools, respectively (C, D).

Supplementary Figure S3

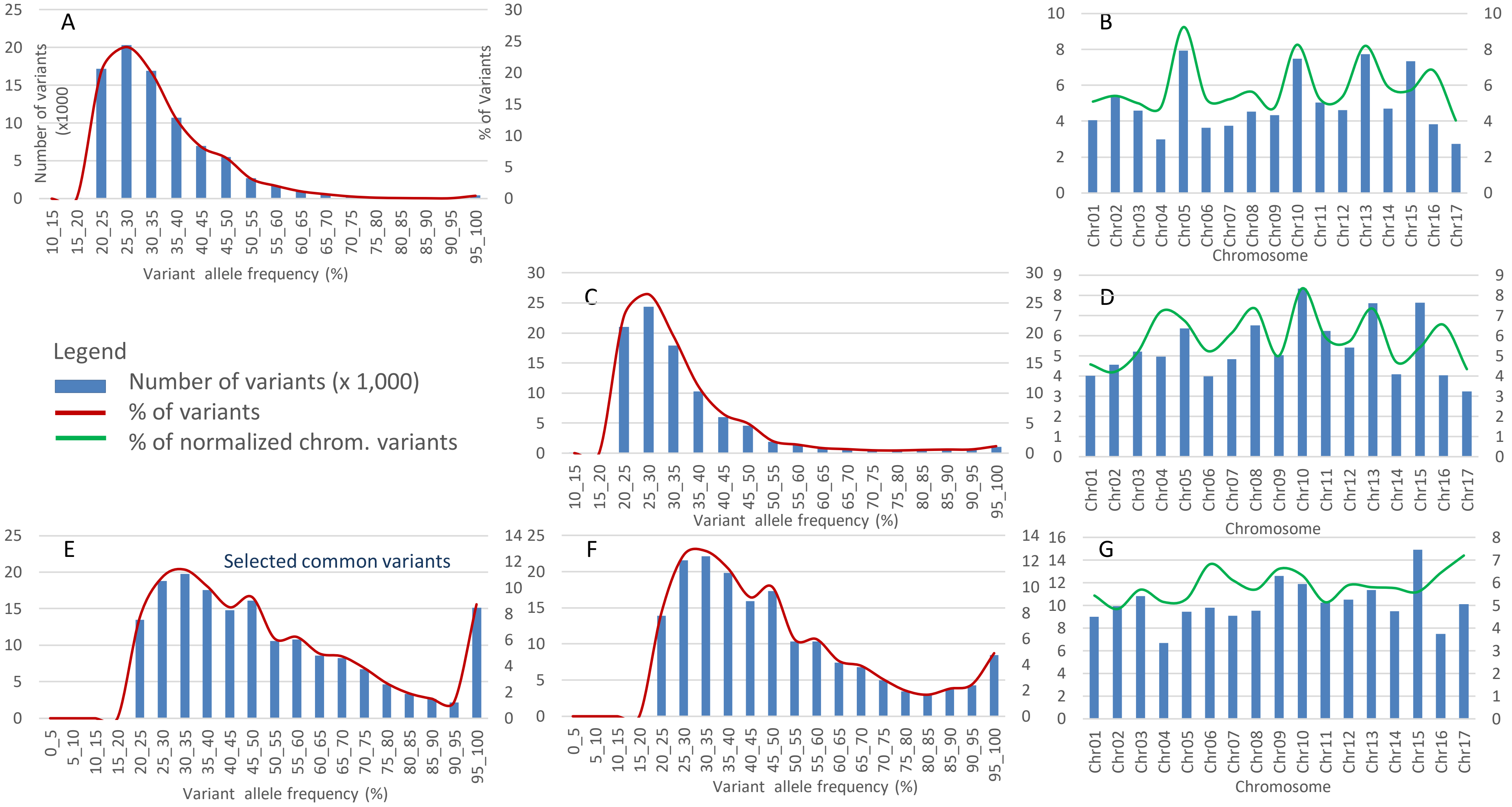

Fig. S3. Distribution of pool specific and common variants. (A-B) Distribution of weeping pool specific variants (84,562) by allele frequency (A) and chromosome (B). (C-D) Distribution of standard pool specific variants (92,148) by allele frequency (C) and chromosome (D). (E-G) Distribution of the common variants (173,169) between the weeping (E) and standard (F) pools by allele frequency, and by chromosome (G). On all charts, the primary vertical axis is for number of variants in 1000s, and the secondary vertical axis is for percent of variants. The percentage of normalized chromosomal variants (shown by the green curves in B, D and G) was calculated by factoring in chromosome physical size so that the data could be directly compared among chromosomes. Two points could be made by examining the variant allele frequency distribution and variant chromosomal distribution: 1) The variants specific to the weeping and standard pools had a highly similar allele frequency distribution with most ranging from 20% to 50% (A, C), suggesting that the selection in establishment of the pools did not lead to much difference between the weeping and standard pools in allele frequency distribution of pool specific variants. The similarities were also shown in the normalized chromosomal distributions with chromosomes 10, 13 and 16 having relatively more variants in both pools (B, D). However, there were more variants on chromosome 5, less on chromosomes 4 and 8 in the weeping pool than in the standard pool. 2) The variants common to both pools had much wider spread of allele frequency, ranging from 20% to 100% (E, F). Although the variants also showed similarities in allele frequency distribution between the two pools, there were markedly more variants (homozygous) of allele frequency 95-100% in the weeping pool (15,143 or 8.7%) than in standard pool (8,446, or 4.9%) (E, F). The normalized chromosomal distributions were largely similar among chromosomes in variants common to both pools, varying narrowly from 4.9% (chromosome 2) to 7.2% (chromosome 17), close to the average distribution of 5.9%, suggesting the number of variants identified from each chromosome was roughly even (G).

Supplementary Figure S4

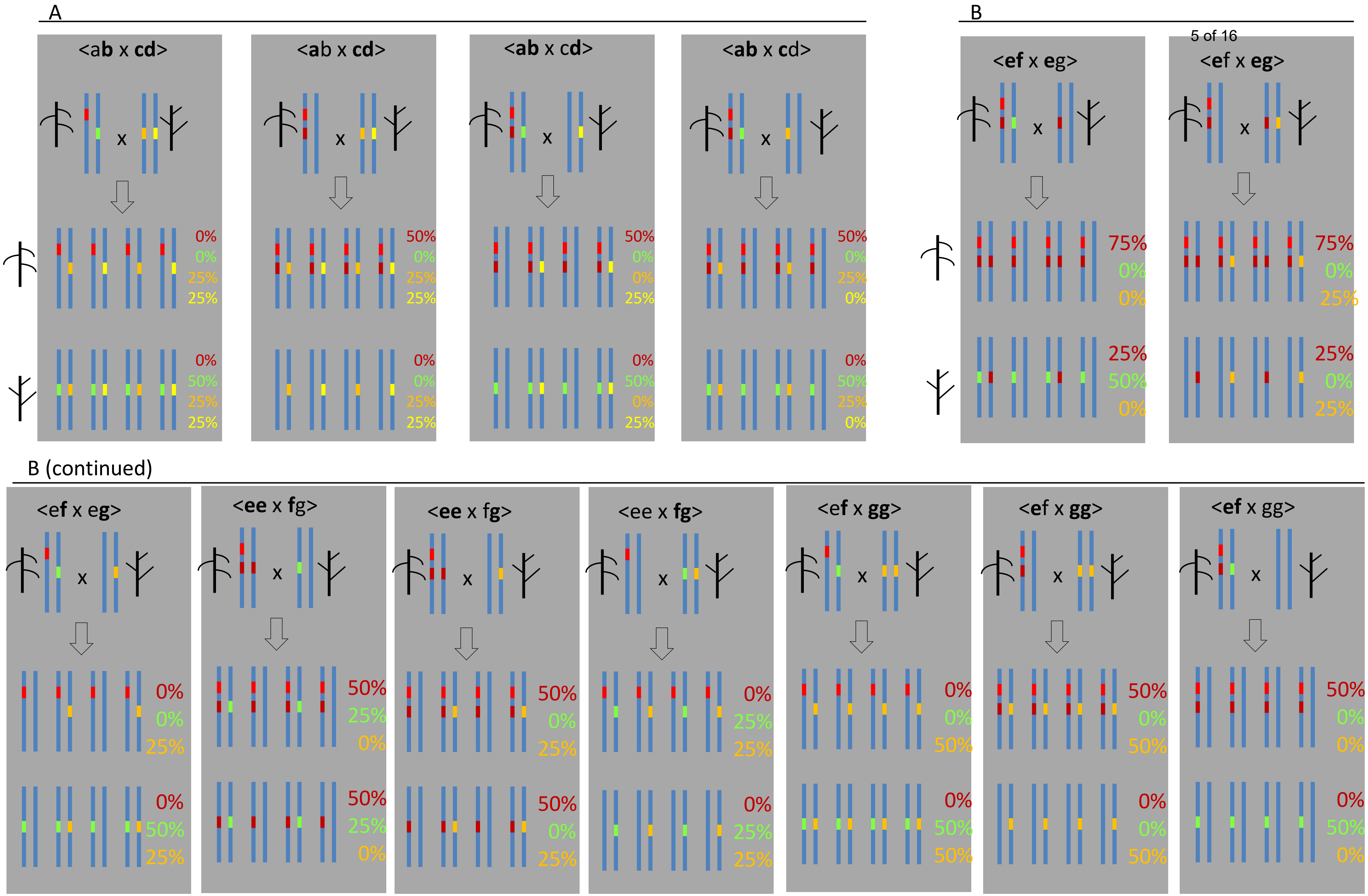

Fig. S4. Schematics for possible segregation types inferred for variant genotype group G5 ‘Complex’. (A) <ab x cd> derived segregation types involving four DNA bases (or three DNA variants). (B) <ef x eg> derived segregation types involving three DNA bases (or two DNA variants). Each segregation type is illustrated in a grey filled rectangular, which includes the two parents at the top, four representative weeping progeny in the mid, and four standard progeny at the bottom. The long vertical lines in blue stand for the chromosomal segment harboring *W*. The short vertical lines in red and in other colors (orange, purple, and green) represent allele *W* and DNA variants in relation to the reference genome, respectively. The tree-like drawings with up- and down-ward ‘branches’ indicate standard and weeping tree phenotypes, respectively. The expected allele frequency of DNA variants in the weeping and standard pools is given and color coded accordingly. In each segregation type denotation, the allele at the first position is designated to be linked to weeping phenotype in the seed parent ‘Cheal’s Weeping’ (e.g. letter ‘a’ in <**ab x cd**>), and those in bold are DNA variants in relation to the apple reference genome (e.g. letters ‘a, c and d’ in <**ab x cd**>).

Supplementary Figure S5

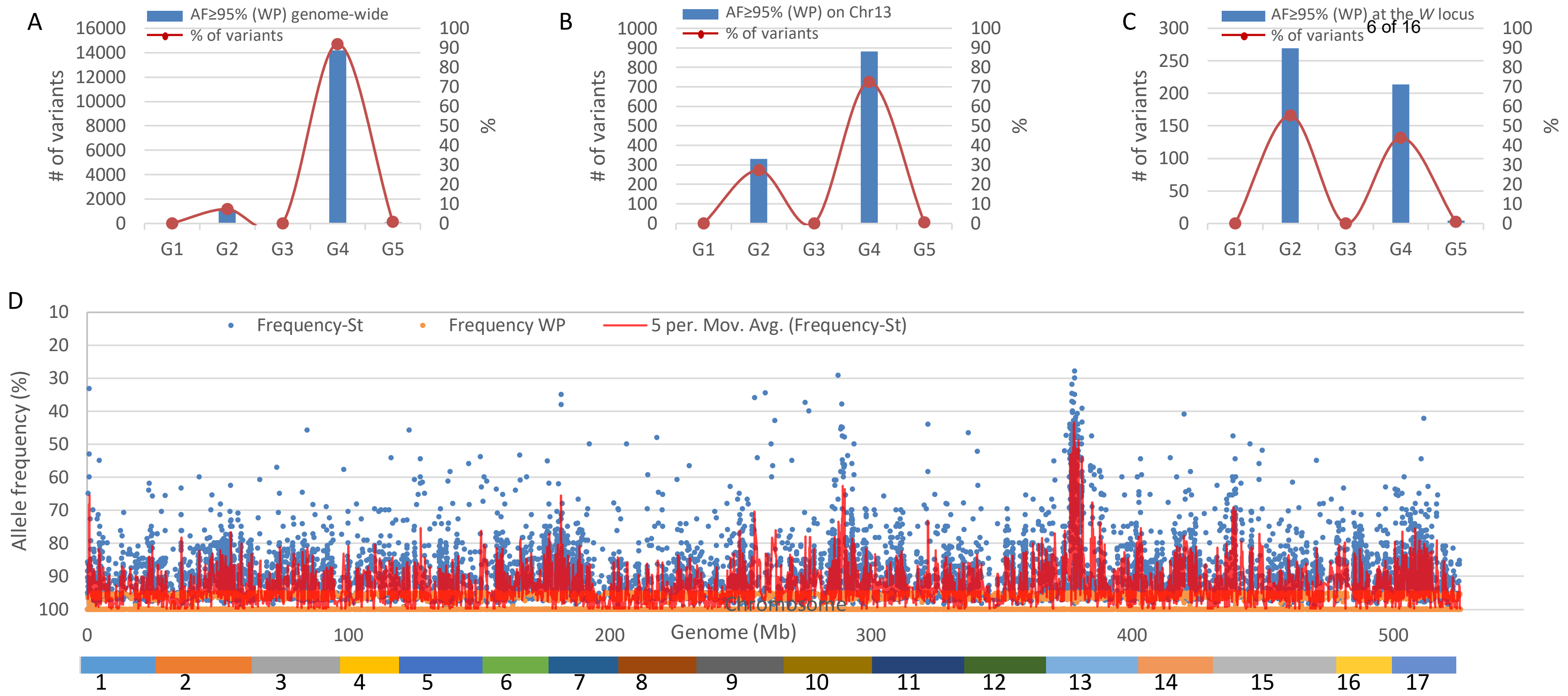

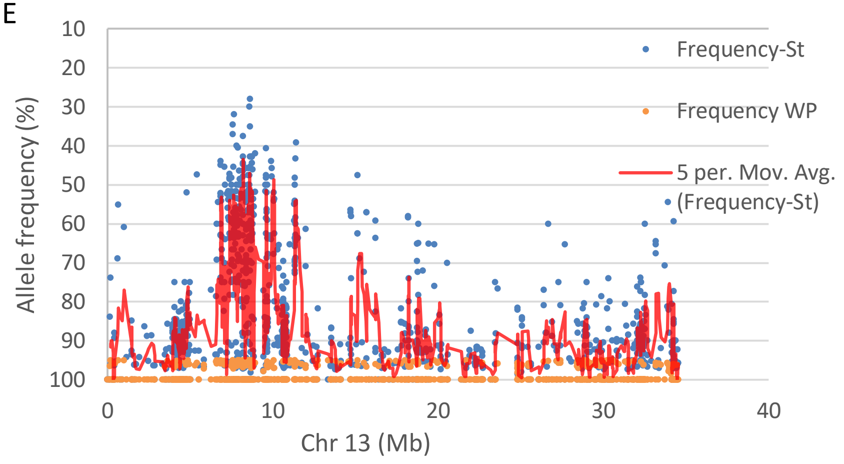

Fig. S5. Evaluating the role of segregation type <**lm** x **ll**> (Type-II variants) in AFDDD mapping using common variants (15,425) selected of allele frequency ≥95% in the weeping pool (WP). (A-C) Number and frequency (%) of such selected variants observed in the five genotype groups at the genome scale (A), on chromosome 13 (B) and in the *W* region (C). G1: heterozygous in weeping / heterozygous in standard (He-W/He-S); G2: Ho-W/He-S; G3: He-W/Ho-S; G4: Ho-W/Ho-S; and G5: 'Complex'. (D-E) Allele frequency distribution of the variants in the weeping and stand pools in the genome (D) and on chromosome 13 (E). The color bar represents the assembled reference genome of 17 chromosomes as numbered. <u>Notes of analysis:</u> The composition of the 15,425 variants was dominated by homozygous variants in G4 of segregation type <**qq** x **qq**>, which accounted for 92.0% (14,185) (A). The second category was the supposed-to-be targeted genotype group G2 by the selection, weighed 7.3% (1,121). Although the weight of this variant genotype group was low, it represented an increase of 135.0% when compared with 3.1% prior to the selection (see Fig. 7D). Plotting the 15,425 variants from both pools against the genome uncovered that the distribution of variants on chromosome 13 was most distinctive from those on any other chromosomes (D). There were 1,219 variants on chromosome 13 (B, E) and 487 in the 7-Mb region of *W* (C). Among these two sets of variants, the frequency of the targeted G2 genotype group were drastically increased to 27.2% (332/1,219) and 54.0% (269/487), respectively (B, C). Examining the 269 variants in the standard pool revealed that there were 79 of allele frequency close to the expected 50%, ranging from 45% to 55% (E). Since the allele frequency range from 45% to 55% differs at least by 40 percentage points from the allele frequency threshold ≥95% used for selection in the weeping pool and there were only 682 variants of AFDD≥40 percentage points in the G2 genotype group genome-wide, the finding of these 79 variants in the *W* region (E) represented a highly significant increase (z=14.1, p=0) of such expected allele frequency profile for Type-II variants in the *W* region of 7-Mb, providing convincing evidence that Type-II variants are an essential part in AFDDD mapping.

Supplementary Figure S6

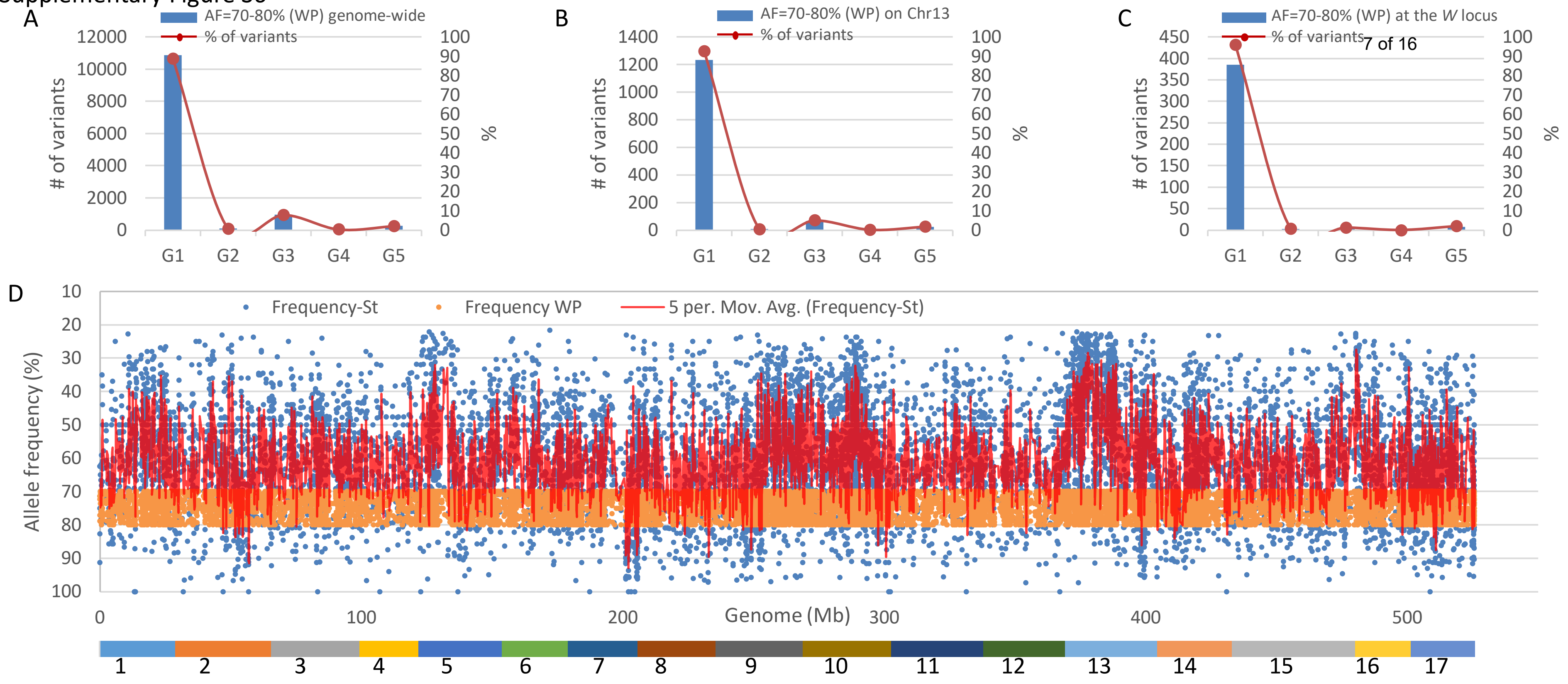

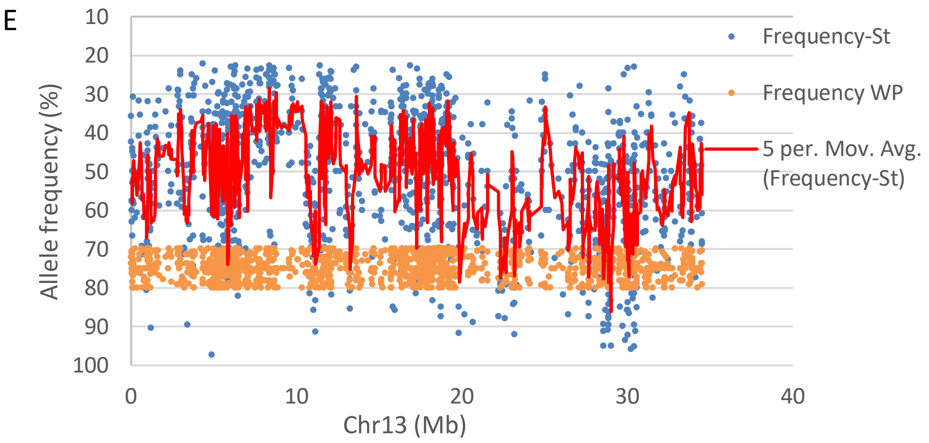

Fig. S6. Evaluating the role of segregation type <**h**k x **h**k> (Type-III variants) in AFDDD mapping using common variants (12,219) selected of allele frequency ranging from 70% to 80% in the weeping pool (WP). (A-C) Number and frequency (%) of such selected variants observed in the five genotype groups at the genome scale (A), on chromosome 13 (B) and in the *W* region (C). G1: heterozygous in weeping / heterozygous in standard (He-W/He-S); G2: Ho-W/He-S; G3: He-W/Ho-S; G4: Ho-W/Ho-S; and G5: 'Complex'. (D-E) Allele frequency distribution of the variants in the weeping and stand pools in the genome (D) and on chromosome 13 (E). The color bar represents the assembled reference genome of 17 chromosomes as numbered. Notes of analysis: Among the 12,219 variants, 89.0% (10,869/12,219) fell in the genotype group G1 (A), suggesting that the selection in the weeping pool not only included Type-III variants, but also led to a 5.5-percentage-point increase from 83.5% in the 173,169 variants (see Fig. 7D). Plotting the 12,219 variants from both pools against the reference genome highlighted that there was a clustered group of 58 variants of allele frequency from 20% to 30% (close to the expected 25%) in the 7-Mb region of *W* on chromosome 13 (D, E). Since the allele frequency threshold ≤30% differs at least by 40 percentage points from the 70%-80% range used for selection in the weeping pool and there were only 908 variants of the G1 genotype group with AFDD≥40 percentage points genome-wide, these 58 variants represented a significant increase (z=8.1, p=0) in the *W* region. In addition, the frequency of the variants of in G1 was 92.6% (1233/1311) on chromosome 13 (B) and 96.0% (385/401) in the 7-Mb region of *W* (C). This was much higher than the original 83.5% (see Fig. 7D), contrasting the overall decrease trend in AFDDD mapping (see Fig. 7A-C). These observations indicated that Type-III variants were selected preferentially in the *W* region due to pooling, i.e. Type-III variants also played a critical role in AFDDD mapping.

Supplementary Figure S7

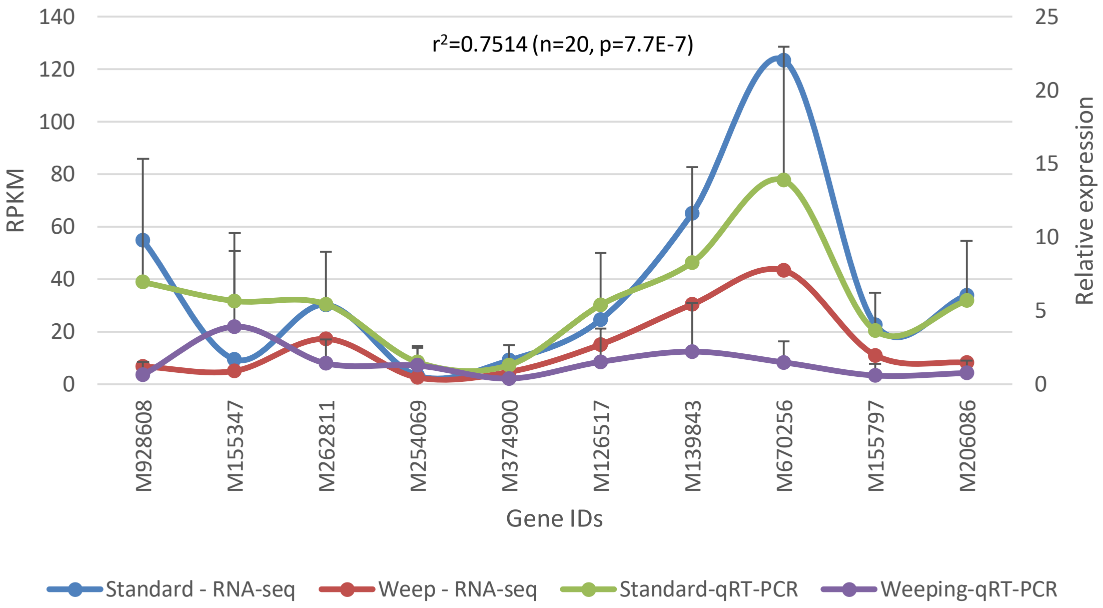

Fig. S7. qRT-PCR validation of gene expression in RNA-seq analysis. The qRT-PCR assays were performed on ten genes in the *W* region. The expression levels of the ten genes in RPKMs in RNA-seq analysis are highly significantly correlated with their relative expression in qRT-PCR assays (r^2=0.7514 (n=20, p=7.7E-7). RPKM: Reads Per Kilobase of transcript per Million mapped reads. The prefix 'MDP0000' in Apple gene Ids (e.g. MDP0000123456) is abbreviated to 'M' (e.g. M123456).

Supplementary Figure S8

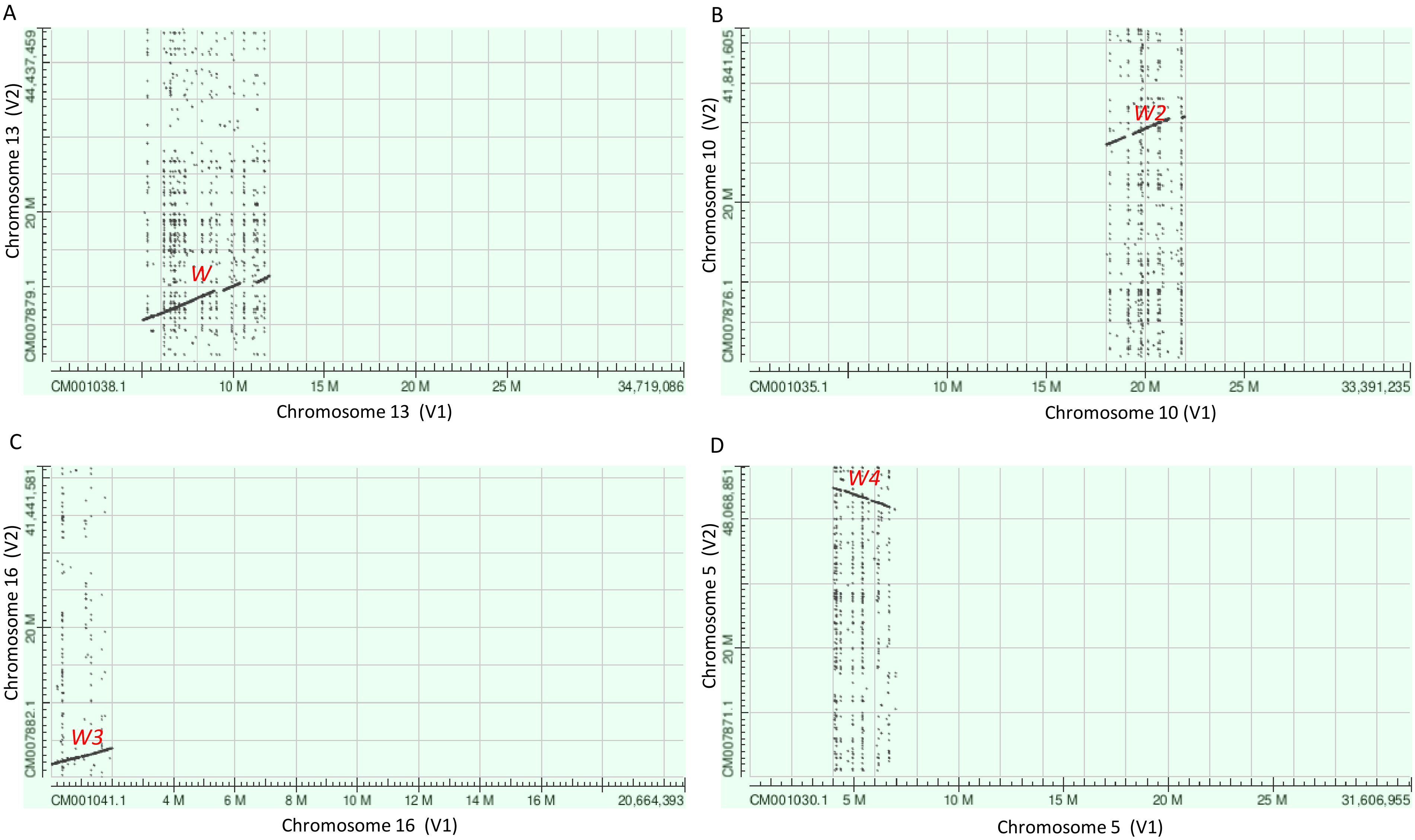

Fig. S8. BLAST-based dot matrix analysis of the genomic regions associated with the weeping trait. The X- and Y-axis and the numbers represent the bases from chromosomes 13 (A), 10 (B), 16 (C) and 5 (D) in the first version (V1, Velasco et al. 2010) and the new version (V2, Daccord et al. 2017) of the apple reference genome, respectively. The *W, W2, W3* and *W4* regions in the V1 and V2 are indicated by dotted lines, which represent genomic regions of high levels of sequence identity between V1 and V2.

Table S1. Reads Mapping Summary

Pools	Reads category	Count	Percentage of reads	Average length	Number of bases	Percentage of bases
Weeping pool	Mapped reads	57,639,266	43.2%	151	8,703,529,166	43.2%
	Not mapped reads	75,677,546	56.8%	151	11,427,309,446	56.8%
	Reads in pairs	29,551,548	22.2%	532.81	4,462,283,748	22.2%
	Broken paired reads	28,087,718	21.1%	151	4,241,245,418	21.1%
	Total reads	133,316,812	100.0%	151	20,130,838,612	100.0%
Standard pool	Mapped reads	66,798,158	44.7%	151	10,086,521,858	44.7%
	Not mapped reads	82,641,060	55.3%	151	12,478,800,060	55.3%
	Reads in pairs	35,549,144	23.8%	514.52	5,367,920,744	23.8%
	Broken paired reads	31,249,014	20.9%	151	4,718,601,114	20.9%
	Total reads	149,439,218	100.0%	151	22,565,321,918	100.0%

Table S2. Variant filtering process

Filters	Weeping	Standard	Common
Total variants: Detection of variants using CLC Genomics Workbench variant detection tools	2,700,059	2,946,289	
Variants non-allelic to reference: Remove variants of reference alleles (including M, R, W, S, Y, K), being hyper allelic or homopolymer	1,306,887 (SNV: 88.5%)	1,380,503 (SNV: 87.8%)	
Pool specific/common variants: Compare the variants between the weeping and standard pools	498,386	573,589	799,089
Putative variants for mapping: Coverage ≥19 (weeping) or ≥20 (standard); Forward/reverse balance: 0.25-0.5. Number of reads with unique start positions: ≥5	84,562	92,148	173,169

Table S3. Primer sequences and their genome physical locations

Primer Name	Marker name/purpose	Sequence (5' to 3')	Targeted positions on chromosomes
Ch13-7641F	Ch13-7641-119bp	TTCGCCTAGTTTGGTCCGTCA	7.641th Mb on chr13
Ch13-7641R		GGGTCCCTGAGAGTCCAGTGC	
Ch13-8181F	Ch13-8181-125bp	TCTTCGAACACACCCGCAAA	8.181th Mb on chr13
Ch13-8181R		GGTTAATGCGCACCGGGTTA	
Ch13-8547F	Ch13-8547-168bp	CCGACCCCAAATGCGTTTAT	8.547th Mb on chr13
Ch13-8547R		GTCCCTGAATTATTCACCACAA	
Ch13-9530F	Ch13-9530-175bp	TTTCTCCGTCCATGTCCTTGA	9.530th Mb on chr13
Ch13-9530R		CCATGGTTGTACTGCGTTTTC	
Ch10-19768F	Ch10-19768-190bp	TTTGGGTCTCCAAATGCATAG	19.768th Mb on chr10
Ch10-19768R		GCTATGTTCAGCTCGTACCG	
Ch10-20017F	Ch10-20017-220bp	GGAATGTTTTGAGTGGTGTCA	20.017th Mb on chr10
Ch10-20017R		GGGAGGGGTGAAGATTCAGT	
Ch13_7923F	SNP validation	CCTTCCGTCTATACCCAGCA	7923174, **7923460**, 7923530 on chr13
Ch13_7923R		TTGAACTCGGATGCAAATCA	
Ch13_8209F	SNP validation	TCGATGAAATTTGCTGTGAAA	**8209678**, **8210175 on chr13**
Ch13_8209R		TTCTCCAAAACTGAGGCAAA	
Ch13_8374F	SNP validation	CTACAGGGAAACCGCTCAAG	8374569, 8375098, 8375311, 8375431, 8375828, 8375948 on chr13
Ch13_8374R		AGCAAGCAAACCATCCTTGT	
Ch13_8758F	SNP validation	GAGCACGGGTTATGGAAGAA	8758311, 8759078, 8759167 on chr13
Ch13_8758R		GCACTGCACGTAATCAAACG	

[1]Positions in bold also were shown in Fig. 8

Table S4. qRT-PCR primer sequences and their targeted gene IDs

Gene IDs	Primer sequences (F/R)	Prod size
MDP0000928608	CACGTGTTCCTTCACGATGT / GCTAACGGGCCAAATATCCT	300
MDP0000155347	GGCGAATCTGTACCAGGAAA / ACGCATAGTTCAACCGGAAA	296
MDP0000262811	CATTCAACAGGCCAACAATG / AAGAAGAAGATGGCCACAGC	300
MDP0000374900	GGACATCCCTTGGTGAGCTA / TCTTGGTTTGGTTCCTTTCG	303
MDP0000126517	GGGAAATGGGTTGTTTTCCT / TTCCCCAATGAAGGACTCTG	299
MDP0000254069	GGGAACAGATCGAACAGAGC / TTTTTGCCTCCCCTTTTCTT	299
MDP0000139843	CTCCAAATCCCAATTCCAGA / GGTGCCGTTGTAGAAAATCG	301
MDP0000670256	ATGGCTTCGAGTTCTGCAAC / CCAAGCTCATTGATCCTTTTC	241
MDP0000155797	CCGGTTGCTATCTGGTTTGT / TCAAGGCCATCTTCTCGTCT	301
MDP0000206086	TCCATATGCTCCACCACAGA / GGATGCAGCCAAATACCACT	305

Table S5. Genotype groups of variants common to both pools and variant segregation types inferred

Genotype group	Variant genotypes observed[a]			Inferred[b]								Notes
	W pool	S pool	# of variants (freq.)	Segregation types (genotype of parents)[c]	W pool genotype		W pool mean AF (%)	S pool genotype		S pool mean AF (%)	AFD between W and S pools (%)	
G1	He-W	He-S	144,558 (83.5%)	<**h**k x **h**k>	hh	hk	75	hk	kk	25	50	Informative for *W*
				<h**k** x h**k**>	hh	hk	25	hk	kk	75	-50	Informative for *w*
				<**nn** x **n**p>	nn	np	75	nn	np	75	0	Not informative
				<nn x n**p**>	nn	np	25	nn	np	25	0	Not informative.
				<**hh** x kk>	hk	hk	50	hk	hk	50	0	Not informative
				<hh x **kk**>	hk	hk	50	hk	hk	50	0	Not informative
G2	Ho-W	He-S	5,353 (3.1%)	<**l**m x **ll**>	ll	ll	100	ml	ml	50	50	Informative for *W*
				<l**m** x ll>	ll	ll	0	ml	ml	50	-50	Informative for *w* using S-pool specific variants
G3	He-W	Ho-S	2,104 (1.2%)	<l**m** x **mm**>	lm	lm	50	mm	mm	100	-50	Informative for *w*
				<**l**m x mm>	lm	lm	50	mm	mm	0	50	Informative for *W* using W-pool specific variants
G4	Ho-W	Ho-S	16,963 (9.8%)	<**qq** x **qq**>	qq	qq	100	qq	qq	100	0	Not informative
G5	complex	complex	4,191 (2.4%)	<**e**f x e**g**>	complex			complex				Complex
				<**ab** x **c**d>	complex			complex				Complex

[a] Homozygous (Ho): variant allele frequency (AF)>80%; Heterozygous (He): 80%> AF>15%; [b] for variants in the *W* region. [c] The alleles in each first position are designated to be linked to weeping phenotype in seed parent 'Cheal's Weeping' and those in bold are a polymorphic variant in relation to the apple reference genome, which are present in both parents. If the variants are from the seed parent 'Cheal's Weeping', they can be linked to the weeping phenotype in either

coupling phase or repulsion phase. W: weeping; S or w: standard; AFD: allele frequency difference. Notes for inferring segregation types for variants in genotype groups G1-G3: G1 (He-W/He-S) was inferred to be caused by six possible variant segregation types, including <**h**k x **h**k>, <h**k x** h**k**>, <**nn** x **n**p>, <nn x n**p**>, <**hh** x kk> and <hh x **kk**>. Of these, the first segregation type <**h**k x **h**k> is the only one that would be informative for mapping allele *W* as it would confer an average '**h**' allele frequencies 75% and 25% in the weeping and standard pools, respectively, allowing a directional (positive) 50-percentage-point difference in allele frequency relative to that in the weeping pool (see also Fig. 3A). The second segregation type <h**k x** h**k**> is informative for the standard phenotype (allele *w*) for a similar but negative 50-percentage-point difference in allele frequency between the weeping and standard pools (see also Fig. 3A). Since this segregation type is not useful for mapping allele *W*, variants of segregation type <h**k x** h**k**> were excluded from further analysis. The latter four were non-informative for mapping as seed parent 'Cheal's Weeping' is homozygous and equal levels of allele frequencies (25%, 50% or 75%) are expected between the two pools, but they were likely the major source for heterozygous variants in the pools based on their expected allele frequencies and the distribution of what actually was observed (see also Fig. S3A, C, E, F). G2 (Ho-W/He-S) was inferred with two possible segregation types <**l**m x **ll**> and <l**m** x ll>. The former, <**l**m x **ll**> is informative for mapping alleles *W* as the allele frequency of '**l**' is expected to be 100% in weeping pool, and 50% in the standard pool, also allowing a positive 50-percentage difference in allele frequency (see also Fig. 3B). The latter, <l**m** x ll> is informative for allele *w* as it would give the allele frequency of '**m**' 0% in the weeping pool, and 50% in the standard pool, leading to a negative 50-percentage difference in allele frequency. It could be used for variants specific to the standard pool, but similar to <h**k x** h**k**>, variants of segregation <l**m** x ll> may not be helpful for mapping allele *W*. G3 (He-W/Ho-S), which is opposite to G2, was similarly inferred with two possible segregation types <**l**m x mm> and <l**m** x **mm**>. In this case, the former would confer a 50% average '**l**' allele frequency in weeping pool specific variants (0% in the standard pool), making segregation type <**l**m x mm> informative for mapping allele *W* (see also Fig. 3C). The latter <l**m** x **mm**> would produce a negative 50-pecentage-point difference in '**m**' allele frequency (50% in weeping, 100% in standard). Again, similar to <h**k x** h**k**>, variants of segregation type <l**m** x **mm**> were disregarded for mapping allele *W*.

Table S6. Summary of RNA-seq reads mapping

RNA-seq sample	Reads category	Apple Reference Genome V1[a] No. of Reads	Apple Reference Genome V1[a] % of clean reads	Apple Reference Genome V2[b] No. of Reads	Apple Reference Genome V2[b] % of clean reads
Standard	Clean reads	52,423,649	100	52,423,649	100
	Mapped reads	39,552,212	75.5	39,606,583	75.6
	mapped uniquely	31,665,782	60.4	37,644,378	71.8
	mapped non-uniquely	7,886,430	15	1,962,205	3.7
	Unmapped reads	12,871,437	24.6	12,817,066	24.5
	Low quality reads	3,802,514		3,802,514	
	rRNA reads	2,932,563		2,932,563	
	Total	59,158,726		59,158,726	
Weeping	Clean reads	39,916,890	100	39,916,890	100
	Mapped reads	30,269,137	75.8	30,558,185	76.6
	mapped uniquely	24,305,524	60.9	28,973,044	72.6
	mapped non-uniquely	5,963,613	14.9	1,585,141	4.0
	Unmapped reads	9,647,753	24.2	9,358,705	23.5
	Low quality reads	2,031,399		2,031,399	
	rRNA reads	1,260,009		1,260,009	
	Total	43,208,298		43,208,298	

[a]Apple Reference Genome V1: Velasco et al. 2010 and Bai et al 2014; [b]Apple Reference Genome V2: Daccord et al 2017

Tables S7-S11 listed below are provided in a Microsoft Excel file:

Table S7. List of expressed genes in the *W* region

Table S8. List of expressed genes in the *W2* region

Table S9. List of expressed genes in the *W* region according to the new reference genome (Daccord et al. 2017)

Table S10. List of expressed genes in the *W2* region according to the new reference genome (Daccord et al. 2017)

Table S11. Differentially expressed genes (DEG) and genes of interest in the *W* and *W2* regions according to both versions of the apple reference genome.

Supplementary References:

Bai Y, Dougherty L, Xu K. 2014. Towards an improved apple reference transcriptome using RNA-seq. *Mol Genet Genomics* **289**, 427-438.

Daccord N, Celton J-M, Linsmith G, Becker C, Choisne N, Schijlen E, van de Geest H, Bianco L, Micheletti D, Velasco R, Di Pierro EA, Gouzy J, Rees DJG, Guerif P, Muranty H, Durel C-E, Laurens F, Lespinasse Y, Gaillard S, Aubourg S, Quesneville H, Weigel D, van de Weg E, Troggio M, Bucher E. 2017. High-quality de novo assembly of the apple genome and methylome dynamics of early fruit development. *Nat Genet* **49**, 1099-1106.

Velasco R, Zharkikh A, Affourtit J et al. 2010. The genome of the domesticated apple (*Malus* x *domestica* Borkh.). *Nature Genetics* **42**, 833-839.
